# Assaying the Effect of Levodopa on the Evaluation of Risk in Healthy Humans

**DOI:** 10.1371/journal.pone.0068177

**Published:** 2013-07-03

**Authors:** Mkael Symmonds, Nicholas D. Wright, Elizabeth Fagan, Raymond J. Dolan

**Affiliations:** 1 Wellcome Trust Centre for Neuroimaging, Institute of Neurology, University College London, London, United Kingdom; 2 Nuffield Department of Clinical Neurosciences, University of Oxford, John Radcliffe Hospital, Oxford, United Kingdom; Inserm, France

## Abstract

In humans, dopamine is implicated in reward and risk-based decision-making. However, the specific effects of dopamine augmentation on risk evaluation are unclear. Here we sought to measure the effect of 100 mg oral levodopa, which enhances synaptic release of dopamine, on choice behaviour in healthy humans. We use a paradigm without feedback or learning, which solely isolates effects on risk evaluation. We present two studies (n = 20; n = 20) employing a randomised, placebo-controlled, within-subjects design. We manipulated different dimensions of risk in a controlled economic paradigm. We test effects on risk-reward tradeoffs, assaying both aversion to variance (the spread of possible outcomes) and preference for relative losses and gains (asymmetry of outcomes - skewness), dissociating this from potential non-specific effects on choice randomness using behavioural modelling. There were no systematic effects of levodopa on risk attitudes, either for variance or skewness. However, there was a drift towards more risk-averse behaviour over time, indicating that this paradigm was sensitive to detect changes in risk-preferences. These findings suggest that levodopa administration does not change the evaluation of risk. One possible reason is that dopaminergic influences on decision making may be due to changing the response to reward feedback.

## Introduction

Risk is a key concept in decision theory, describing situations of uncertainty where actions lead to a range of possible outcomes [1]. Risk is ubiquitous both in the natural world and in complex human economies, and many decisions can be conceptualised as a trade-off between risk and potential reward. Risk perception is driven by multiple features of a decision, such as the spread (measured as variance) of possible outcomes [2], [3], [4], as well as asymmetry between better or worse than average outcomes (measured as skewness) [5], [6], [7]. While some individuals prefer to take a risk in exchange for higher possible reward, ‘risk-averse’ individuals require a greater financial incentive to make a risky choice (i.e. have a greater ‘risk-premium’). Dopamine plays a central role in reward and risk-based decision-making, but its specific contribution to risk evaluation in healthy humans is unclear.

Clinically, Parkinson’s disease (PD), where nigro-striatal dopamine pathways are impoverished, is associated with disrupted decision-making [8], [9]. Dopamine agonists, used to treat PD, can cause pathological gambling behavior [10], [11], a side-effect exacerbated in dual therapy with both dopamine agonists and levodopa [12], although some studies report no effect of dopamine augmentation therapy on decision making in PD patients [13]. Manipulation of dopamine levels in rats disrupts decision-making under uncertainty in foraging tasks. Amphetamine (which augments dopamine release), D1-, or D2-receptor agonists can increase preference for a risky choice, and the effects of amphetamine can be abolished by dopamine receptor blockade [14]. Neuroimaging studies have identified subcortical and cortical dopaminoceptive regions linked to risk and choice [15], [16], [17], [18]. Single-unit recording studies show that tonic firing of dopaminergic midbrain neurons scales with risk [19], although there is debate about whether this signal represents prediction errors during learning [20], [21], encoded initially at the time of reward feedback and following learning at the time of reward-predictive signals [22].

Previous studies have not addressed which specific aspect of risk dopaminergic modulation might influence. For example, dopamine has been proposed to act as a generic neuromodulatory signal encoding the uncertainty of predictions [23], hence might be expected to have a specific impact on evaluation of variance. Dopamine agonists have also shown to systematically alter choices and neural activity following better than average (i.e. unexpectedly good) rewards, hence dopamine might alternatively impact upon the evaluation of relative gains versus losses in a choice (skewness). Moreover, previous tasks have tended to employ continual reward feedback, either in the context of learning or for gambling tasks where risk is explicit. Dopamine release is strongly associated with reward feedback and reward anticipation [24], therefore it is possible that dopamine alters the response to feedback as well as affecting the statistical evaluation of risk *per se*. Performance impairments following dopaminergic depletion in tasks involving risk assessment, such as the Iowa Gambling Task (IGT) [25], could be explained in terms of any of these effects.

Here we assessed the effect of dopamine administration on the statistical evaluation of risk, measuring decision-making in healthy subjects performing two different economic gambling experiments. In the first experiment, we tested whether dopamine influences the trade-off between (expected) reward and variance. In the second experiment, we tested if dopamine influences the evaluation of variance, or of relative gains and losses (skewness). Crucially, we employed a task where we could independently manipulate both expected value-variance and variance-skewness. Critically, all choices are based on explicit presentations of risky gambles and no feedback is given during the task, which controls for effects on reward feedback or learning such that we can measure solely effects upon the evaluation of risk. Additionally, using behavioural modeling we were able to determine whether dopamine simply makes choices more random (which can often appear as a tendency towards risk-neutrality). Using a randomised, double blinded, placebo controlled administration of L-dopa (which increases vesicular dopamine release in the central nervous system) [26], we aimed to detect any systematic effects of dopamine augmentation upon individuals’ risk-reward trade-offs.

## Materials and Methods

### 1. Ethics Statement

Experiments were approved by the Institute of Neurology (University College London) Ethics Committee. All subjects gave fully informed written consent for participation.

### 2. Setup

In total forty healthy participants were recruited, (Experiment 1∶7 male/13 female, mean age 23.1, SD = 3.44, range 19–32; Experiment 2∶7 male/13 female, mean age 22.4, SD = 4.27, range 18–33) and attended on two separate occasions one week apart. On each week, subjects received either a 100 mg dose of L-dopa (Madopar - levodopa/benserazide, 100/25 mg, Roche) dissolved in fruit squash or an indistinguishable fruit squash placebo, administered 50 minutes before behavioral testing to allow dopamine to reach peak plasma and neural concentration [27]. Stimuli were presented and responses recorded using Cogent presentation software (Wellcome Trust Centre for Neuroimaging, London) written in MATLAB (version 6.5, MathWork, Natick, MA). The task was performed on a standard PC, and choices were indicated using a keyboard. We provided a 5-minute practice tutorial to demonstrate the paradigm. Data were analysed using MATLAB and SPSS (SPSS for Windows, Rel. 12.0.1. 2003. Chicago: SPSS Inc.).

### 3. Paradigms

**Figure 1:**
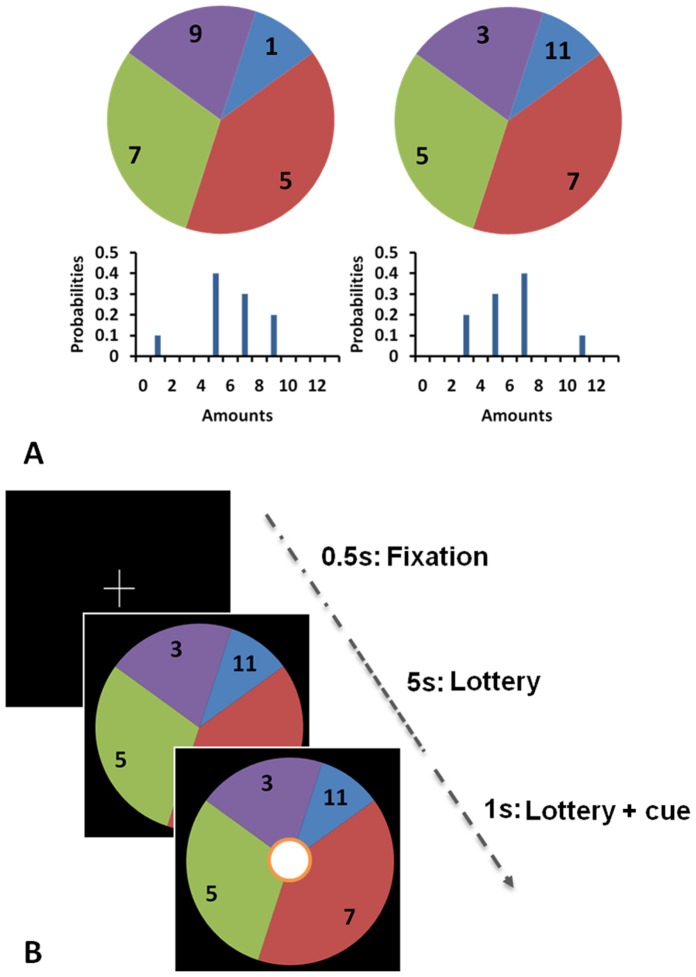
Experimental Paradigm. A. We represented gambles on-screen as pie-charts. The pie chart was divided into different segments showing possible outcomes from the lottery. The numbers written in each segment showed the monetary value of each outcome (in pounds sterling) and the angle subtended by each segment indicated the probability of each outcome occurring. A negatively skewed gamble (left) has a small chance of a worse than average outcome (the tail of the distribution is to the left). Conversely, a positively skewed gamble (right) has a small chance of a better than average outcome (the tail is to the right). Both example gambles have identical expected value (£6) variance (5£^2^), but opposite skewness (+/−7.2£^3^). B. Each task consisted of 252 trials. For each trial, a pie chart was shown, and after 5 seconds, a cue to respond appeared on screen (for 1 second). Subjects indicated by a button press while the cue was on-screen if they wanted to gamble on the lottery, or alternatively select a fixed, sure amount of money (of £4.50 throughout). At the end of the experiment on their second visit, four trials were randomly selected and played out for real. If subjects had elected to gamble, we resolved the lottery by an on-screen graphic of a red ball spinning around the outside of the pie which stopped at a randomly selected position.

To dissociate preferences for different components of risk, in terms of dispersion (variance) and asymmetry of outcomes (skewness), we implemented a previously-used decision-making task design [28] that controls the distribution of outcomes to ensure that expected value, variance and skewness of a set of lotteries could be manipulated independently by design. We tested whether dopamine administration influenced the impact of either average return, the spread of outcomes (measured as variance) or relative loss and gain (asymmetry of outcomes, measured as skewness). On each trial, participants were required to choose (5s decision time) between taking a ‘sure’ (fixed) amount of money or electing to ‘gamble’ (choosing to play a lottery with a number of potential outcomes). Gambles were represented as 4-segment pie-charts (**Figure 1**). We conducted two separate experiments, each with 20 subjects and an identical structure, apart from the specific stimulus sets employed.

#### 3.1 Experiment 1: Independent manipulation of expected value and variance

We constructed a stimulus set of 252 lotteries where expected value and variance were independent and varied over a range (**see File S1**). Expected value of the lotteries ranged from £3.25 to £8.00, while variance ranged from 0.47 to 24.05£^2^. All stimuli were symmetric (i.e. had zero skewness). Stimuli were constrained to have 4 outcomes (segments of the pie chart), with outcome probabilities varying in minimum 0.1 increments between 0 and 1. These restrictions allow the generation of a space of possible lotteries varying in expected value and variance. EV and variance were orthogonal by design (correlation coefficient: *r* = 0.07).

#### 3.2 Experiment 2: Independent manipulation of variance and skewness

Here, we constructed a stimulus set of 252 lotteries where variance and skewness were independent and varied over a range (**see File S2**). Expected value of the lotteries was constant (£5.95–£6.05). Variance ranged from 1.7 to 30.9£^2^. Skewness ranged from −38.6 to 38.6£^3^. Variance and skewness were orthogonal (correlation coefficient: *r* <0.01). As in experiment 1, stimuli were constrained to have 4 outcomes (segments of the pie chart), with outcome probabilities varying in minimum 0.1 increments between 0 and 1.

This setup allows us to test whether dopamine has a systematic influence on the trade-off between risk and reward in the absence of decision feedback or not. We anticipated that we might detect non-specific effects, such as a shift in risk preference from week 1 to week 2 or an effect of drug on choice randomness, hence our paradigm and analysis enables us to distinguish and quantify these effects separately from effects on risk dimensions. If dopamine purely affects the evaluation of anticipated (mean) reward, we would expect to observe a shift in risk-reward tradeoff in experiment 1 but not in experiment 2 where expected value is constant. If dopamine alternatively affects just the evaluation of relative losses and gains in a gamble (which we operationalise here as skewness), then we would expect to observe solely an effect in experiment 2. If dopamine affects the encoding of variance, we would expect effects in both experiment 1 and in experiment 2.

On each occasion, the participant made decisions about the same set of 252 choices. Using a diverse spread of lotteries enables us to map out responses (choices) to stimuli representing an entire array of risk and value combinations. Consistent tradeoffs between different dimensions of value, variance, and skewness can then be explored and tested by comparing the performance of different decision-making models where subjects express preferences for each of these components. In addition, utilising a large range of possible gambles is akin to psychophysical methods [29], and means that any specific biases engendered by the configuration of a particular gamble will have a minor influence on an overall decision making metric. On each occasion, choices were presented in a randomised order, and the orientation and ordering of pie chart segments was also randomised on each trial. Stimulus sets were constructed and the sure amount alternative was fixed at £4.50, such that participants would choose to gamble approximately 50% of the time on average (based on pilot studies). This meant that the stimulus sets had the greatest power to distinguish subtle effects on changes in EV-variance and variance-skewness tradeoffs.

### 4. Payment

To ensure that subjects chose in accordance with their genuine preferences, payment was incentive compatible. Four trials were selected randomly (two from participants’ first session and two from their second session) and played out for real at the end of the second visit. For each selected trial, if subjects chose the sure amount, they won £4.50, whereas if they elected to gamble, the lottery was resolved with an animated ‘roulette wheel’ graphic of a red ball spinning around the pie chart, before coming to rest at a randomly selected position which determined their winnings from that trial. Winnings ranged from £20.00 to £42.50 (mean £32.23), including a baseline participation fee of £12.

### 5. Behavioural Modelling

For a given lottery with 4 potential outcomes *(m_1_, m_2_,… m_N_)*, with probabilities *p = p_1_, p_2_, …p_n_*, we define the statistical moments (expected value (EV), variance (Var), skewness (Skw)) of the outcome distribution as follows:
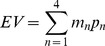
(1)

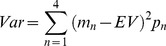
(2)

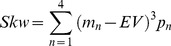
(3)


We analysed choice data by fitting a linear mean-variance-skewness model (**MVS**) where individuals are allowed to express different preferences for variance and skewness. To establish whether individuals are indeed responding to risk, we test this **MVS** model against a series of reduced models, where decisions are based on mean difference (**M**) alone (where subjects only take account of the difference between the sure amount and the expected value of the gamble in selecting actions), or a mean-variance model (**MV**).

We then define the subjective value, or utility (U) of each lottery for our models:

Mean model (**M**)
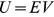
(4)


Mean-variance model (**MV**)
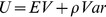
(5)


Mean-variance-skewness model (**MVS**)
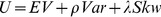
(6)


ρ and λ are free parameters, ρ reflecting preference for variance, and λ reflecting preference for positive versus negative skewness respectively.

We test a further model able to account for different preferences for positive and negative skewness (**MVS2**).
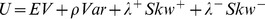
(7)Where Skw^+^ indictates Skw≥0 and Skw^−^ indicates Skw<0, and λ^+^ and λ^−^ reflect preferences for positive and negative skewness respectively.

Our models compare the utility of the lottery with the value of the sure amount (*S*) to generate a trial-by-trial probability of choosing the lottery over the sure amount, using a logistic/softmax function which allows for noise in action selection (by free parameter β).
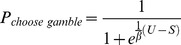
(8)


We estimated best-fitting model parameters using maximum likelihood analysis, with unconstrained optimisation implemented with a non-linear Nelder-Mead simplex search algorithm in Matlab (Matlab, Natwick, USA) and compared models using the Bayesian Information Criterion (BIC) [30] as an approximations to the model evidence and penalising model complexity [31].

## Results

### 1. Behaviour

#### 1.1 Experiment 1: Expected value versus variance trade-off

**Figure 2:**
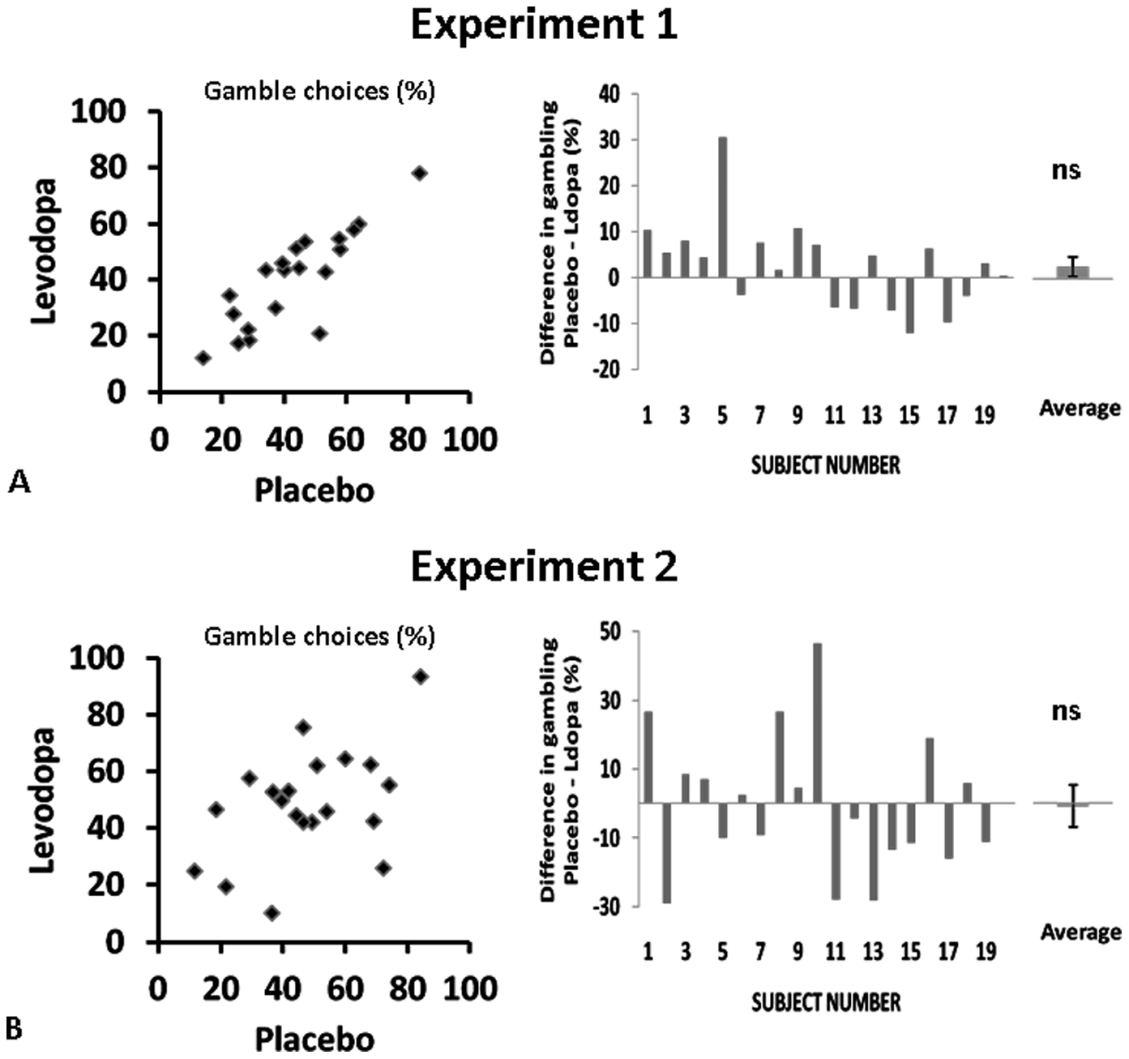
Behavioural results. A. Experiment 1– Expected value – variance manipulation. B. Experiment 2– Variance – skewness manipulation. On left, scatterplots of percentage gambling choices on levodopa and placebo (n = 20). Gambling choice percentage is very highly correlated for individuals for the two separate (placebo and levodopa) attended sessions (linear fit through origin - Experiment 1: F_1,19_ = 431, p<0.01, r = 0.98; Experiment 2: F_1,19_ = 123, p<0.01, r = 0.93).On right, percentage differences in gambling choice between placebo and levodopa conditions plotted per subject with average effect size (ns = non-significant, error bars show standard error).

Subjects distributed their choices between gamble and sure options throughout the course of the experiment (as intended by experimental design), choosing to gamble on average 43.1% (SD ±17.23%) of trials on placebo, and 40.6% (SD ±17.15%) on L-dopa (compared to a risk-neutral decision maker who would have gambled on 79% of trials with this stimulus set). There was no significant difference between these proportions (paired t-test, t_19_ = 1.20, p = 0.24) (**Figure 2A**). Even accounting for the effect of order in a 2×2 repeated measures ANOVA (drug/placebo × drug week 1/drug week 2), there was no overall effect of drug administration (F_1,9_ = 0.05, p = 0.95), although the order effect reached trend significance (F_1,9_ = 4.38, p = 0.06), with an average −5.7+/−8.0% change in percentage gambling from week 1 to week 2.

#### 1.2 Experiment 2: Variance versus skewness trade-off

Here, subjects also distributed their choices between gamble and sure options throughout the course of this experiment, choosing to gamble in on average 47.8% (SD ±19.5%) of trials on placebo, and 48.4% (SD ±19.3%) on L-dopa (compared to a risk-neutral decision maker who would have always gambled with this stimulus set). There was no significant difference between these proportions (paired t-test, t_19_ = 0.15, p = 0.88) (**Figure 2B**). As above, we also entered data into a 2×2 repeated measures ANOVA, which confirmed no overall effect of drug administration (F_1,9_ = 0.03, p = 0.87), although the order effect again approached significance (F_1,9_ = 3.83, p = 0.07), with an average −8.1+/−17.9% change in percentage gambling from week 1 to week 2.

We further examined for any effects on risk in specific subdomains (e.g. only for positively skewed gambles with a small chance of high rewards). Choice data were partitioned into 4 domains - high/low variance and positive/negative skewness decisions - and entered into repeated measures ANOVA. This revealed no significant effect of drug (F_1,9_ = 0.01, p = 0.91), with no significant interaction between drug and domain (F_3,9_ = 0.11, p = 0.96).

### 2. Behavioural Modelling

We next performed a model-based analysis of participants’ choices, where we estimated parameters for variance- and skewness-aversion from an economic decision model. This model based analysis was designed firstly to test whether subjects’ made consistent choices, trading risk and reward in a coherent manner (as opposed to being insensitive to risk). A lack of drug effect could be purely due to a baseline indifference to risk in our subjects. Secondly, parameters estimated from a behavioural model have greater sensitivity to detect small systematic changes in risk-preferences than a simple summary analysis of percentage gambling. Thirdly, we can test specific behavioural hypotheses by comparing the performance of different models in explaining the data. Dopamine may induce a change in risk-preference (i.e. the trade-off between risk and potential reward) or may simply induce a general bias in choice that leads to an increased predilection for gambling, without altering risk-sensitivity. Finally, we can also test whether dopamine significantly changed choice randomness or noise as we explicitly model this as a free parameter that determines the slope of the logistic (softmax) function.

#### 2.1 Sensitivity to risk

We independently manipulated variance and skewness, and predicted that individuals’ preferences would be sensitive to both aspects of risk. To test this, we compared a set of models which predict choice on the basis of either the mean value of sure vs lottery options alone (model **M**, risk-insensitive), the mean and lottery variance (model **MV**), and for experiment 2, the mean variance and skewness (model **MVS**).

**Figure 3:**
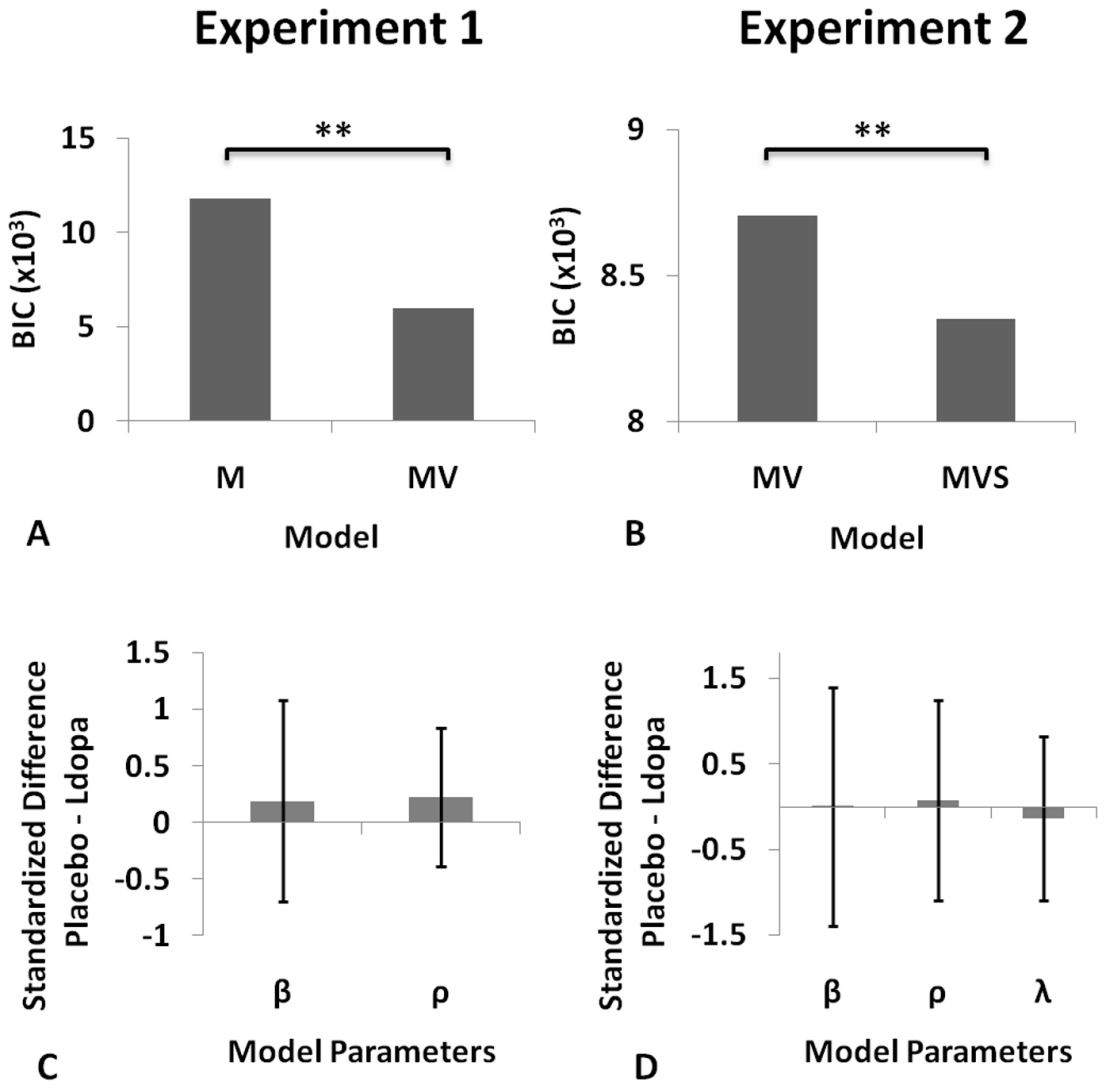
Behavioural modelling. A. Experiment 1: Log-evidence, approximated by the Bayesian information criterion (BIC), for mean only (**M**) and mean-variance (**MV**) models. Fixed effects analysis of Group Bayes Factors shows **MV** highly significantly superior to **M** model (likelihood ratio test: p<10^−5^). B. Experiment 2: BIC scores for mean-variance (**MV**) and mean-variance-skewness (**MVS**) models. **MVS** highly significantly superior to **MV** model (likelihood ratio test: p<10^−5^). BIC* = k.ln(n) –2ln(L),* where *L* is the model likelihood, *n* is the number of observations and *k* is the number of free parameters. Lower BIC indicates better model fit. C. Experiment 1: Differences in (standardised) model parameters for choice noise (β) and variance preference (ρ) between placebo and levodopa sessions. Error bars show standard deviation. D. Experiment 2: Differences in (standardised) model parameters for choice noise (β), variance (ρ), and skewness (λ) preference between placebo and levodopa sessions. Error bars show standard deviation.

Risk-sensitive models far outperformed the risk-insensitive model demonstrating that subjects’ choices were significantly influenced by decision risk (**Figure 3A**). Moreover, the **MVS** model was superior to the **MV** model in predicting choice in experiment 2 (**Figure 3B**), showing that individuals are influenced by both risk dimensions of variance and skewness (experiment 1: BIC**_M_** = 11827, BIC**_MV_** = 5964, **MV** model posterior probability>0.99 (very strong evidence in favour of **MV** model); experiment 2: BIC**_M_** = 12999, BIC**_MV_** = 8708, BIC**_MVS_** = 8352, **MVS** model posterior probability>0.99 (very strong evidence in favour of **MVS** model)).

We estimated parameters corresponding to tastes for our 2 independent risk domains, variance (ρ) and skewness (λ), as well as choice randomness (β), for each subject and each condition (drug/placebo) independently. We entered individually estimated parameters into group-level analysis to test for differences. Participants were on average averse to variance in both experiment 1 (ρ_PLACEBO_ = −0.13, SD +/−0.08; ρ_LDOPA_ = −0.15, SD +/−0.10) and experiment 2 (ρ_PLACEBO_ = −0.11, SD +/−0.07; ρ_LDOPA_ = −0.12, SD +/−0.10). While 19/20 subjects here were variance averse, skew preferences were heterogeneous (λ_PLACEBO_ = 0.0039, SD +/−0.0253; λ_LDOPA_ = 0.0011, SD +/−0.0169), with 8/20 positive skew-seeking individuals (on placebo). β values were low (experiment 1: β_PLACEBO_ = 0.52 SD +/−0.17, β_LDOPA_ = 0.49 SD +/−0.13; experiment 2: β_PLACEBO_ = 0.53 SD +/−0.18, β_LDOPA_ = 0.53 SD +/−0.24), indicating that choices were well partitioned by the behavioural models and that choice noise was low.

#### 2.2 Drug-induced changes in preference

There were no significant differences in ρ, λ, or β parameters between drug and placebo sessions, either in experiment 1 (paired t-tests, ρ: t_19_ = 1.63, p = 0.12; β: t_19_ = 0.35, p = 0.96; **Figure 3C**) or in experiment 2 (paired t-tests, ρ: t_19_ = 0.29, p = 0.78; λ: t_19_ = 0.62, p = 0.54; β: t_19_ = 0.01, p = 0.99, **Figure 3D**). This indicates that L-dopa had no effect on altering risk-return tradeoffs, either in terms of the impact of the spread of outcomes, or on relative losses and gains. Moreover, there was no effect on choice randomness – choices were equally noisy in both sessions.

We also checked for evidence of skewness intransitivity in experiment 2 by fitting a different model (**MVS2**) where subjects can express different sensitivities for positive and negatively skewed lotteries. If individuals prefer or dislike both positive and negative skew, this will not be well captured by the **MVS** model, hence lowers our power to detect drug-induced changes in preference. Indeed, the **MVS2** model was significantly better than the simpler **MVS** model at explaining choice, even accounting for its extra parameter (BIC**_MVS_** = 8352, BIC**_MVS2_** = 7914), and revealed that 9/20 participants disliked both positive and negative skew compared to symmetric gambles, while 3/20 preferred both positive and negative skew. However, even in this more sensitive model, we detected no difference in preference (or choice noise) between drug and placebo (average parameter values: ρ_PLACEBO_ = −0.12, SD +/−0.08, ρ_LDOPA_ = −0.12, SD +/−0.10; λ^+^_PLACEBO_ = 0.0052, SD +/−0.0207; λ^+^_LDOPA_ = 0.0073, SD +/−0.0246; λ^−^_PLACEBO_ = −0.0027, SD +/−0.0241; λ^−^_LDOPA_ = −0.0002, SD +/−0.0339; β_PLACEBO_ = 0.55 SD +/−0.23, β_LDOPA_ = 0.53 SD +/−0.23. Paired t-tests, ρ: t_19_ = 0.21, p = 0.84; λ^+^: t_19_ = 0.50, p = 0.62; λ^−^: t_19_ = 0.35, p = 0.73; β: t_19_ = 0.30, p = 0.77).

#### 2.3 Changes in preference over time

Given the observed (non-significant) changes in percentage gambling over time in both experiments, we tested whether this translated into significant changes in model parameter estimates for risk preference, expected if the model-based approach is indeed more sensitive in detecting such changes. As anticipated, there were significant changes in variance preference parameters from week 1 to week 2 (paired t-tests: experiment 1 - ρ: t_19_ = 2.82, p = 0.01; experiment 2 - ρ: t_19_ = 2.09, p = 0.05; λ: t_19_ = 1.59, p = 0.13, **Figure S1**).

## Discussion

We explored the effect of L-dopa administration on evaluation of different aspects of risk, and the impact on decision making, in healthy humans. To control for possible drug-induced changes in learning and response to reward, our paradigm was specifically designed to isolate effects on risk evaluation. Moreover, by using an economic task and behavioural modelling, we could empirically quantify changes in risk preferences.

### 1. Risk Sensitivity

All subjects were sensitive to risk, being generally averse to increasing variance (spread of outcomes), and with a range of preferences for skewed gambles (asymmetrical distribution of outcomes). Our behavioural modelling revealed the importance of both risk dimensions, as simpler models based only on the average anticipated reward (expected value) failed to explain behaviour as well as the **MV** and **MVS** models. Crucially, we find that L-dopa administration does not affect preferences for either variance or for skewness. Moreover, the fact that we observe no changes in experiment 1 means that neither average value, variance, or the trade-off between them is influenced by L-dopa.

Our paradigm substantially differs from previous psychopharmacological studies of risk, as we assess choice preferences for independent statistical features of a distribution of outcomes. This economic quantification of risk preference lends power and precision over previous paradigms used to assess risk-taking (e.g. Cups task [32], Game of Dice Task [33], Risk Task [34]). In these tasks risk is explicitly described for participants, as opposed to less specific alternatives such as the IGT and Ballon Analogue Risk Task (BART) [35] where participants are uncertain about the real probability of rewards. Summary measures of percentage gambling are used to delineate risk-taking, however these measures are specific to the set of stimuli used - stimulus sets in which different features, such as expected value, variance, and skewness, are often correlated. This renders it difficult to quantify precise effects, to map these on to specific psychological or neural processes, and to determine the true effect size of choice shifts.

Our observed lack of drug effect cannot be simply accounted for by an inability of the task to engage participants, or due to the large variety of risk levels presented impairing the ability to make appropriate responses. The fact that subjects demonstrated clear and consistent risk-sensitivity (to both variance and skewness) indicates that subjects were attentive and able to execute the task correctly (i.e. to make considered decisions between risky and risk-free choices in accordance with intrinsic preferences). There was also a strong consistency in individuals’ choices over time. This correlation was slightly less in experiment 2, perhaps reflecting that preferences for variance are more stable than those for skewness. Here we employed a within-subject design with twenty subjects tested on two separate occasions, which potentially could induce a desire for consistency in choice behavior and explain a lack of effect. However, this paradigm and closely related tasks with similar sample sizes have been used to delineate changes in risk-preference induced by physiological manipulations such as hunger and satiety [36] and psychological manipulations such as making decisions for losses versus gains [37].

Our paradigm was sensitive to small changes in risk preference, as revealed by the systematic decrease in propensity to gamble from week 1 to week 2 across subjects. Moreover, the model-based analysis showed greater sensitivity in detecting this change than the overall measure of percentage gambling choice. The 6% choice shift in experiment 1 was explained by a significant increase in variance aversion (ρ), which translates in financial terms to a difference in risk premium of £0.80 for a 50∶50 chance of winning £10 or £0 (i.e. this same gamble becomes £0.80 less appealing to an individual from week 1 to week 2). Thus although our paradigm was sensitive to this small, systematic drift in risk attitude over time, we did not detect a drug effect.

It can also be difficult to distinguish between drug induced changes in risk-reward tradeoff versus changes in choice noise in previous paradigms. Inattentive or random responding in a binary choice task will shift choice proportions towards 50%, an effect which can often masquerade as a change in risk evaluation, and may account for some previous findings in drug studies [38]. Importantly, we can account for non-specific effects of drug administration on the randomness of responses in our behavioural model, which partitions effects into changes in risk preferences, and the independent quantification of choice noise.

### 2. Effects of L-dopa

It is possible that our L-dopa administration did not cause sufficient physiological effects to influence behaviour, and inter-individual variability in drug pharmokinetics is a potential source of heterogeneity in our results. However, the oral dose of 100 mg L-dopa used here has been previously employed in a range of studies, demonstrating effects on semantic priming [39], [40], [41], cognitive control [42], learning and memory [43], [44], [45], [46], perception [47], and decision-making [48], [49]. A 100 mg oral dose minimises side effects of nausea or drowsiness, which can significantly impact upon performance. Although oral administration is used in standard clinical practice, this delivery method relies on systemic absorption into the central nervous system which is another source of variability. We also ensured a delay between drug administration and task execution such that the task was performed at the expected time of peak L-dopa concentration [50], making it unlikely that the lack of effect is only due to low L-dopa levels.

### 3. Risk Versus Reward Learning

Given previous reports of the effects of dopamine manipulation on risk-taking in patients and healthy humans, we speculate about alternative explanations for the lack of effect here. We were careful to design our task to eliminate effects of learning and reward feedback. Here, our stimuli were explicit, whereas in many previous studies the level of risk associated with a stimulus needs to be learnt over a number of trials. For example, in the IGT where different decks of cards are presented, the quality of the decks needs to be ascertained by repeated sampling [51]. Since dopamine has a central role in reward-based learning, and encodes reward-prediction error [22], it is possible that the effects of dopaminergic manipulation are expressed at this early stage when probabilistic contingencies are being acquired. L-dopa augments dopamine release at synapses [26] and could encourage risk-taking by boosting the apparent value of stimuli in the face of unpredictable reward, which has been proposed as a neurocomputational mechanism underlying addiction [52]. Moreover, differential effects of dopamine on the response to rewards and punishments [53], [54], [55] which could encourage risk-taking if positive prediction errors are given more weight than negative prediction errors [56], [57], leading to an overestimation of value for risky stimuli. This effect cannot be engendered in our paradigm, as all choices were resolved after the end of the experiment, as standard in experimental economic paradigms [58].

Related to this is the distinction between explicit risk, where the probabilities of outcomes are precisely known, and ambiguity, where the outcome distribution is unknown and needs to be learnt through exploration. Ambiguity-aversion is a well-known behavioural bias [59], distinct from risk attitude [60], however the two are conflated in risk-taking tasks such as the BART and IGT where risks are not explicit. Our finding of a lack of effect of dopamine on risk evaluation is consistent with findings in PD patients of specific deficits in decision making under ambiguity rather than risk [61].

### 4. Dopaminergic Effects, Genetic Variability, and the Role of other Neurotransmitters

Other explanations for the absence of a change in risk-taking with L-dopa include specific dopamine receptor subtype effects, genetic heterogeneity in responses, or other neurotransmitter systems being central to risk evaluation.

Impulse control disorders are noted side effects of dopamine agonists in particular [10]. Risk-taking may therefore be a specific by-product of D1-receptor stimulation, an effect opposed by simultaneous D2-receptor stimulation. Contrary to this is the finding that the risk-promoting effects of amphetamine are abolished by both D1- and D2-receptor antagonists [14], and that D1, D2 and D3-specific dopamine agonists are all reported to induce gambling behaviour [62], [63], although D3- receptor agonist effects are also reported to decrease risky choice in an animal model [14]. L-dopa itself potentiates gambling behaviour in co-administration with dopamine agonists [12]. An abnormal baseline in PD patients with depleted nigrostriatal systems may engender disrupted dopamine receptor expression or sensitivity that render this vulnerability to agonist effects, however these side-effects have also been reported in individuals treated for restless legs syndrome [64]. L-dopa promotes the phasic and tonic release of dopamine from synapses in response to afferent depolarisation, while dopamine agonists enhance the tonic stimulation of post- and pre-synaptic receptors in a non-physiological manner [65]. Thus, differential effects of these agents could be attributed to a distinction between phasic and tonic dopamine, which have been suggested to map onto different computational processes [66]. An important future avenue for research is to delineate whether effects on gambling behaviour are dopamine-receptor specific, and whether any effects pertain to risk evaluation or other processes such as learning and reward-responsiveness.

Genetic heterogenetiy may also determine the effects of L-dopa on risk-taking, given that polymorphisms in the D1 [67], [68], D2 [69], [70] and D4 [71], [72] receptor, and the dopamine transporter gene [72], have been associated with risky or impulsive behaviour. Although we were careful to consider within-subject effects to minimise the role of between-subject variability, it is still possible that significant variability could mask an effect at an individual level. For example, pharmacogenetic interactions have been demonstrated, with a report of L-dopa increasing risk-taking, in a paradigm with feedback and dynamic risk changes, only for subjects with a specific DRD4 polymorphism [73]. Dopamine receptor polymorphisms are also suggested to mediate different neuronal responses to reward during gambling tasks [74], [75], [76]. Given the constellation of findings, and the fact that individual polymorphisms appear to account for only a small fraction of the tendency to pathological gambling [72], the specific effects of each on different elements of the decision making and learning process remains a challenge for future large scale investigations.

It is also possible that an alternative neurotransmitter is involved in imbuing risk-preference. Evidence from both neuroimaging and single-unit recording studies have implicated serotonin in reward processing [77], [78], [79], and serotonin augmentation [80] or depletion [81], [82] alters reward and risk-based decision-making. An effect of serotonin on risk-attitude could also contribute to the effect of satiety and starvation on decision making under risk [36]. While the rewarding and appetitive effects of food have been attributed to dopaminergic systems [83], serotonin is also critical in behavioural homeostasis [84]. Serotonin and dopamine receptor genes may also interact to determine propensity for risk-taking [85].

### Conclusion

The central finding from this study is that 100 mg L-dopa administration does not affect risk preference in healthy humans. This is in contrast to studies implicating dopamine in risky decision making, and potentially suggests that these effects of dopamine are effected via other mechanisms such as modulation of learning or response to reward, rather than the evaluation of risk itself. Given that a lack of effect cannot be conclusively interpreted as evidence of no effect, these conclusions are necessarily speculative. Clearly future work is needed to test the hypotheses arising from the present study, specifically that dopamine influences decision-making by affecting the response to feedback rather than risk evaluation. It also remains to be determined if this lack of effect is specific to levodopa or a generic finding about dopaminergic transmission. Our paradigm offers careful control over different aspects of risk which are often conflated in behavioural studies, and a quantification of risk-preference independent of non-specific effects on choice noise due to attentional changes. Thus, this task could be further adapted to dissociate possible effects of dopamine on reward feedback, and to explore the effects of stimulation of different dopamine receptor subtypes as well as other neuromodulatory agents.

Economically inspired paradigms can offer experimental control to selectively manipulate aspects of a decision and sensitively assay pharmacological effects. Understanding the role of dopamine in decision making under risk is critically important given its central role as a signalling agent in the brain, the cognitive effects of disease process affecting the dopaminergic system such as Parkinson’s disease, and side effects from clinical treatments as well as drugs of abuse.

## Supporting Information

Figure S1 **Changes in risk preference between weeks.** Differences in (standardised) risk preference model parameters between weeks, plotted per subject with average effect size (error bars show standard error, ∗ indicates p≤0.05). A. Experiment 1, showing difference in variance preference (ρ). B. Experiment 2, showing differences in variance (ρ), and skewness (λ) preferences. On average, subjects’ behaviour showed greater aversion to variance between week 1 and week 2.
(TIF)

File S1 **Stimulus set, Experiment 1.** Stimulus set of 252 4-outcome lotteries. Expected value of the lotteries ranges from £3.25 to £8.00; variance ranges from 0.47 to 24.05£^2^.
(DOCX)

File S2 **Stimulus set, Experiment 2.** Stimulus set of 252 4-outcome lotteries. Expected value of the lotteries is constant (£5.95–£6.05). Variance ranges from 1.7 to 30.9£^2^. Skewness ranges from −38.6 to 38.6£^3^.
(DOCX)
