# Supplementary figures and images for: Assaying the Effect of Levodopa on the Evaluation of Risk in Healthy Humans
Source: PLoS One. 2013 Jul 3;8(7):e68177. doi: 10.1371/journal.pone.0068177 (PMC3700857; doi:10.1371/journal.pone.0068177)

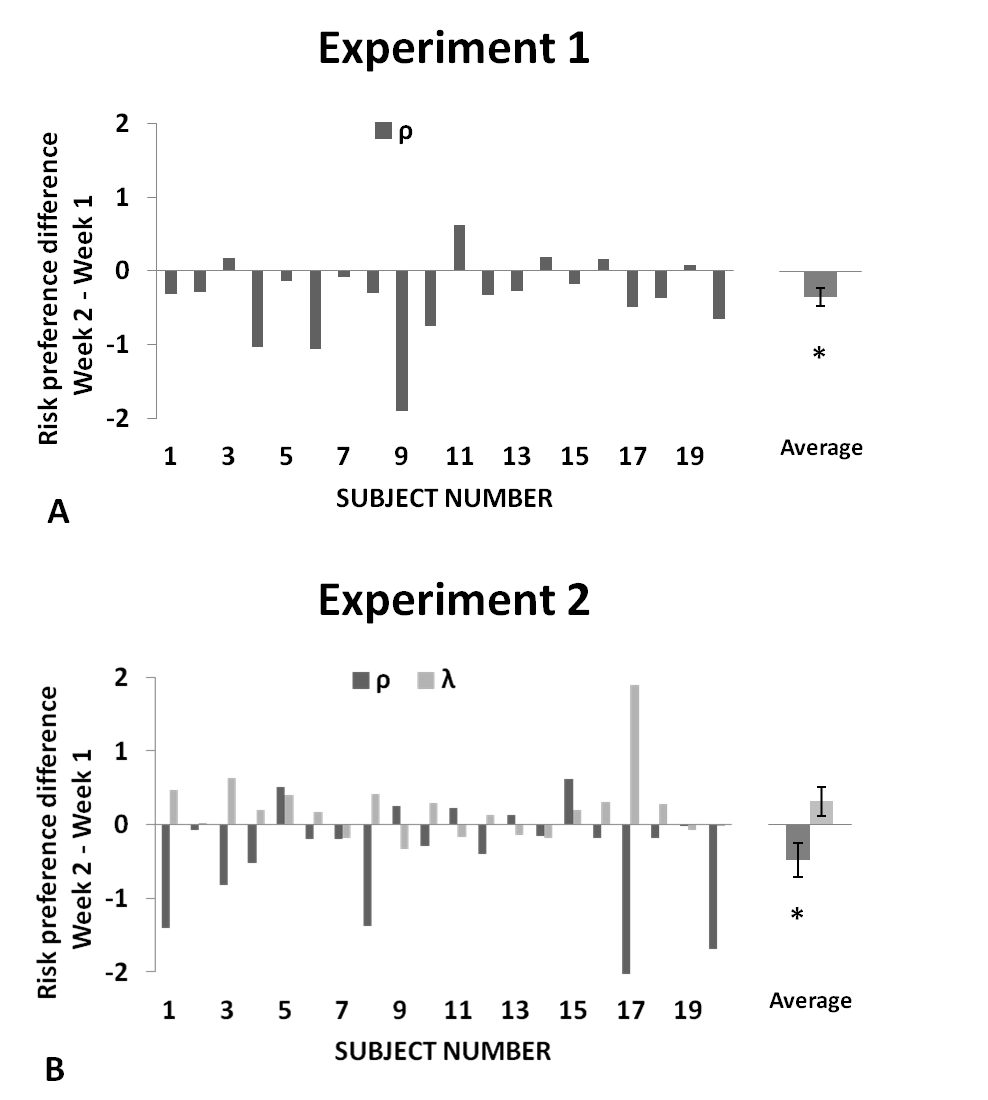

Supplement: Figure S1 — Changes in risk preference between weeks. Differences in (standardised) risk preference model parameters between weeks, plotted per subject with average effect size (error bars show standard error, ∗ indicates p≤0.05). A. Experiment 1, showing difference in variance preference (ρ). B. Experiment 2, showing differences in variance (ρ), and skewness (λ) preferences. On average, subjects’ behaviour showed greater aversion to variance between week 1 and week 2. (TIF) [file pone.0068177.s001.tif]
