# Supplementary material for: Assaying the Effect of Levodopa on the Evaluation of Risk in Healthy Humans
Source: PLoS One. 2013 Jul 3;8(7):e68177. doi: 10.1371/journal.pone.0068177 (PMC3700857; doi:10.1371/journal.pone.0068177)
Supplement: File S1 — Stimulus set, Experiment 1. Stimulus set of 252 4-outcome lotteries. Expected value of the lotteries ranges from £3.25 to £8.00; variance ranges from 0.47 to 24.05£2. (DOCX) [file pone.0068177.s002.docx]

|  | | Probabilities | | | | | Amounts (£) | | | | |  | |  | |
| --- | --- | --- | --- | --- | --- | --- | --- | --- | --- | --- | --- | --- | --- | --- | --- |
| Trial | **p1** | | **p2** | **p3** | **p4** | **m1** | | **m2** | **m3** | **m4** | **EV** | | **Var** | |  |
| 1 | 0.4 | | 0.1 | 0.1 | 0.4 | 3 | | 1.5 | 5 | 3.5 | 3.25 | | 0.66 | |  |
| 2 | 0.1 | | 0.4 | 0.1 | 0.4 | 5.5 | | 2 | 1 | 4.5 | 3.25 | | 2.26 | |  |
| 3 | 0.35 | | 0.15 | 0.35 | 0.15 | 1 | | 4.5 | 5.5 | 2 | 3.25 | | 4.01 | |  |
| 4 | 0.3 | | 0.3 | 0.2 | 0.2 | 6 | | 0.5 | 5 | 1.5 | 3.25 | | 5.76 | |  |
| 5 | 0.2 | | 0.3 | 0.3 | 0.2 | 5 | | 6.5 | 0 | 1.5 | 3.25 | | 7.56 | |  |
| 6 | 0.3 | | 0.2 | 0.2 | 0.3 | 6.5 | | 0.5 | 6 | 0 | 3.25 | | 9.36 | |  |
| 7 | 0.2 | | 0.2 | 0.3 | 0.3 | 6.5 | | 0.5 | 7 | 0 | 3.5 | | 10.95 | |  |
| 8 | 0.4 | | 0.1 | 0.1 | 0.4 | 0 | | 0.5 | 6.5 | 7 | 3.5 | | 11.60 | |  |
| 9 | 0.15 | | 0.35 | 0.15 | 0.35 | 5 | | 4 | 2.5 | 3.5 | 3.75 | | 0.51 | |  |
| 10 | 0.1 | | 0.4 | 0.4 | 0.1 | 6 | | 2.5 | 5 | 1.5 | 3.75 | | 2.26 | |  |
| 11 | 0.35 | | 0.35 | 0.15 | 0.15 | 1.5 | | 6 | 5 | 2.5 | 3.75 | | 4.01 | |  |
| 12 | 0.3 | | 0.3 | 0.2 | 0.2 | 6.5 | | 1 | 2 | 5.5 | 3.75 | | 5.76 | |  |
| 13 | 0.2 | | 0.3 | 0.2 | 0.3 | 7.5 | | 5.5 | 0 | 2 | 3.75 | | 7.46 | |  |
| 14 | 0.3 | | 0.2 | 0.3 | 0.2 | 7 | | 1 | 0.5 | 6.5 | 3.75 | | 9.36 | |  |
| 15 | 0.25 | | 0.25 | 0.25 | 0.25 | 0 | | 6.5 | 7.5 | 1 | 3.75 | | 10.81 | |  |
| 16 | 0.4 | | 0.4 | 0.1 | 0.1 | 0 | | 7.5 | 1 | 6.5 | 3.75 | | 12.76 | |  |
| 17 | 0.15 | | 0.35 | 0.35 | 0.15 | 0.5 | | 7.5 | 0 | 7 | 3.75 | | 13.01 | |  |
| 18 | 0.4 | | 0.1 | 0.4 | 0.1 | 0 | | 7 | 7.5 | 0.5 | 3.75 | | 13.36 | |  |
| 19 | 0.15 | | 0.15 | 0.35 | 0.35 | 3 | | 5 | 3.5 | 4.5 | 4 | | 0.48 | |  |
| 20 | 0.25 | | 0.15 | 0.1 | 0.5 | 2.5 | | 6.5 | 1.5 | 4.5 | 4 | | 2.25 | |  |
| 21 | 0.1 | | 0.4 | 0.4 | 0.1 | 0 | | 5 | 3 | 8 | 4 | | 4.00 | |  |
| 22 | 0.2 | | 0.2 | 0.3 | 0.3 | 3 | | 5 | 1 | 7 | 4 | | 5.80 | |  |
| 23 | 0.2 | | 0.1 | 0.3 | 0.4 | 0 | | 5.5 | 7.5 | 3 | 4 | | 7.50 | |  |
| 24 | 0.25 | | 0.25 | 0.25 | 0.25 | 0.5 | | 1.5 | 7.5 | 6.5 | 4 | | 9.25 | |  |
| 25 | 0.2 | | 0.2 | 0.3 | 0.3 | 1 | | 7 | 7.5 | 0.5 | 4 | | 10.95 | |  |
| 26 | 0.1 | | 0.4 | 0.1 | 0.4 | 3.5 | | 8 | 4.5 | 0 | 4 | | 12.85 | |  |
| 27 | 0.2 | | 0.2 | 0.3 | 0.3 | 0.5 | | 7.5 | 8 | 0 | 4 | | 14.50 | |  |
| 28 | 0.1 | | 0.4 | 0.4 | 0.1 | 7 | | 0 | 8 | 1 | 4 | | 14.60 | |  |
| 29 | 0.35 | | 0.15 | 0.15 | 0.35 | 0 | | 0.5 | 7.5 | 8 | 4 | | 14.88 | |  |
| 30 | 0.1 | | 0.1 | 0.4 | 0.4 | 0.5 | | 7.5 | 0 | 8 | 4 | | 15.25 | |  |
| 31 | 0.35 | | 0.15 | 0.15 | 0.35 | 4.5 | | 5.5 | 3 | 4 | 4.25 | | 0.51 | |  |
| 32 | 0.1 | | 0.4 | 0.4 | 0.1 | 2 | | 3 | 5.5 | 6.5 | 4.25 | | 2.26 | |  |
| 33 | 0.35 | | 0.35 | 0.15 | 0.15 | 6.5 | | 2 | 5.5 | 3 | 4.25 | | 4.01 | |  |
| 34 | 0.3 | | 0.2 | 0.2 | 0.3 | 1.5 | | 2.5 | 6 | 7 | 4.25 | | 5.76 | |  |
| 35 | 0.3 | | 0.3 | 0.2 | 0.2 | 2.5 | | 6 | 8 | 0.5 | 4.25 | | 7.46 | |  |
| 36 | 0.25 | | 0.25 | 0.25 | 0.25 | 3.5 | | 5 | 0 | 8.5 | 4.25 | | 9.31 | |  |
| 37 | 0.2 | | 0.3 | 0.2 | 0.3 | 5 | | 0 | 3.5 | 8.5 | 4.25 | | 11.06 | |  |
| 38 | 0.1 | | 0.4 | 0.4 | 0.1 | 1.5 | | 8 | 0.5 | 7 | 4.25 | | 12.76 | |  |
| 39 | 0.4 | | 0.1 | 0.4 | 0.1 | 8.5 | | 4 | 0 | 4.5 | 4.25 | | 14.46 | |  |
| 40 | 0.25 | | 0.25 | 0.25 | 0.25 | 0 | | 0.5 | 8.5 | 8 | 4.25 | | 16.06 | |  |
| 41 | 0.2 | | 0.3 | 0.2 | 0.3 | 0.5 | | 0 | 8 | 8.5 | 4.25 | | 16.46 | |  |
| 42 | 0.1 | | 0.4 | 0.1 | 0.4 | 7.5 | | 8.5 | 1 | 0 | 4.25 | | 16.56 | |  |
| 43 | 0.15 | | 0.35 | 0.15 | 0.35 | 8 | | 0 | 0.5 | 8.5 | 4.25 | | 16.86 | |  |
| 44 | 0.4 | | 0.1 | 0.1 | 0.4 | 0 | | 8 | 0.5 | 8.5 | 4.25 | | 17.26 | |  |
| 45 | 0.35 | | 0.35 | 0.15 | 0.15 | 5 | | 4 | 5.5 | 3.5 | 4.5 | | 0.48 | |  |
| 46 | 0.1 | | 0.15 | 0.5 | 0.25 | 2 | | 7 | 5 | 3 | 4.5 | | 2.25 | |  |
| 47 | 0.4 | | 0.1 | 0.4 | 0.1 | 3.5 | | 8.5 | 5.5 | 0.5 | 4.5 | | 4.00 | |  |
| 48 | 0.2 | | 0.3 | 0.2 | 0.3 | 3.5 | | 1.5 | 5.5 | 7.5 | 4.5 | | 5.80 | |  |
| 49 | 0.2 | | 0.1 | 0.4 | 0.3 | 0.5 | | 6 | 3.5 | 8 | 4.5 | | 7.50 | |  |
| 50 | 0.25 | | 0.25 | 0.25 | 0.25 | 2 | | 7 | 8 | 1 | 4.5 | | 9.25 | |  |
| 51 | 0.3 | | 0.2 | 0.2 | 0.3 | 8 | | 7.5 | 1.5 | 1 | 4.5 | | 10.95 | |  |
| 52 | 0.1 | | 0.1 | 0.4 | 0.4 | 5 | | 4 | 0.5 | 8.5 | 4.5 | | 12.85 | |  |
| 53 | 0.2 | | 0.2 | 0.3 | 0.3 | 8 | | 1 | 0.5 | 8.5 | 4.5 | | 14.50 | |  |
| 54 | 0.25 | | 0.25 | 0.25 | 0.25 | 9 | | 1 | 8 | 0 | 4.5 | | 16.25 | |  |
| 55 | 0.15 | | 0.15 | 0.35 | 0.35 | 8 | | 1 | 0 | 9 | 4.5 | | 17.85 | |  |
| 56 | 0.1 | | 0.1 | 0.4 | 0.4 | 1.5 | | 7.5 | 0 | 9 | 4.5 | | 18.00 | |  |
| 57 | 0.25 | | 0.25 | 0.25 | 0.25 | 0 | | 9 | 0.5 | 8.5 | 4.5 | | 18.13 | |  |
| 58 | 0.3 | | 0.2 | 0.3 | 0.2 | 9 | | 0.5 | 0 | 8.5 | 4.5 | | 18.55 | |  |
| 59 | 0.4 | | 0.1 | 0.4 | 0.1 | 9 | | 1 | 0 | 8 | 4.5 | | 18.65 | |  |
| 60 | 0.35 | | 0.15 | 0.35 | 0.15 | 9 | | 8.5 | 0 | 0.5 | 4.5 | | 18.98 | |  |
| 61 | 0.4 | | 0.4 | 0.1 | 0.1 | 9 | | 0 | 8.5 | 0.5 | 4.5 | | 19.40 | |  |
| 62 | 0.15 | | 0.15 | 0.35 | 0.35 | 3.5 | | 6 | 4.5 | 5 | 4.75 | | 0.51 | |  |
| 63 | 0.4 | | 0.1 | 0.4 | 0.1 | 6 | | 7 | 3.5 | 2.5 | 4.75 | | 2.26 | |  |
| 64 | 0.15 | | 0.15 | 0.35 | 0.35 | 3.5 | | 6 | 2.5 | 7 | 4.75 | | 4.01 | |  |
| 65 | 0.1 | | 0.1 | 0.4 | 0.4 | 9.5 | | 0 | 6 | 3.5 | 4.75 | | 5.76 | |  |
| 66 | 0.2 | | 0.2 | 0.3 | 0.3 | 8.5 | | 1 | 3 | 6.5 | 4.75 | | 7.46 | |  |
| 67 | 0.25 | | 0.25 | 0.25 | 0.25 | 5.5 | | 4 | 9 | 0.5 | 4.75 | | 9.31 | |  |
| 68 | 0.3 | | 0.2 | 0.3 | 0.2 | 9 | | 5.5 | 0.5 | 4 | 4.75 | | 11.06 | |  |
| 69 | 0.1 | | 0.1 | 0.4 | 0.4 | 2 | | 7.5 | 8.5 | 1 | 4.75 | | 12.76 | |  |
| 70 | 0.1 | | 0.4 | 0.4 | 0.1 | 5 | | 0.5 | 9 | 4.5 | 4.75 | | 14.46 | |  |
| 71 | 0.15 | | 0.15 | 0.35 | 0.35 | 3.5 | | 6 | 9.5 | 0 | 4.75 | | 16.26 | |  |
| 72 | 0.35 | | 0.15 | 0.35 | 0.15 | 9.5 | | 7.5 | 0 | 2 | 4.75 | | 18.06 | |  |
| 73 | 0.2 | | 0.3 | 0.2 | 0.3 | 0 | | 0.5 | 9.5 | 9 | 4.75 | | 19.86 | |  |
| 74 | 0.15 | | 0.35 | 0.15 | 0.35 | 8.5 | | 9.5 | 1 | 0 | 4.75 | | 20.01 | |  |
| 75 | 0.1 | | 0.4 | 0.4 | 0.1 | 8 | | 0 | 9.5 | 1.5 | 4.75 | | 20.16 | |  |
| 76 | 0.25 | | 0.25 | 0.25 | 0.25 | 9 | | 0.5 | 9.5 | 0 | 4.75 | | 20.31 | |  |
| 77 | 0.2 | | 0.3 | 0.3 | 0.2 | 0.5 | | 9.5 | 0 | 9 | 4.75 | | 20.76 | |  |
| 78 | 0.1 | | 0.4 | 0.4 | 0.1 | 1 | | 9.5 | 0 | 8.5 | 4.75 | | 20.86 | |  |
| 79 | 0.15 | | 0.15 | 0.35 | 0.35 | 0.5 | | 9 | 9.5 | 0 | 4.75 | | 21.21 | |  |
| 80 | 0.4 | | 0.4 | 0.1 | 0.1 | 0 | | 9.5 | 9 | 0.5 | 4.75 | | 21.66 | |  |
| 81 | 0.15 | | 0.35 | 0.35 | 0.15 | 4 | | 4.5 | 5.5 | 6 | 5 | | 0.48 | |  |
| 82 | 0.5 | | 0.15 | 0.1 | 0.25 | 5.5 | | 7.5 | 2.5 | 3.5 | 5 | | 2.25 | |  |
| 83 | 0.1 | | 0.1 | 0.4 | 0.4 | 9 | | 1 | 4 | 6 | 5 | | 4.00 | |  |
| 84 | 0.1 | | 0.4 | 0.1 | 0.4 | 10 | | 6 | 0 | 4 | 5 | | 5.80 | |  |
| 85 | 0.3 | | 0.2 | 0.1 | 0.4 | 8.5 | | 1 | 6.5 | 4 | 5 | | 7.50 | |  |
| 86 | 0.25 | | 0.25 | 0.25 | 0.25 | 7.5 | | 8.5 | 1.5 | 2.5 | 5 | | 9.25 | |  |
| 87 | 0.15 | | 0.35 | 0.4 | 0.1 | 10 | | 2 | 7 | 0 | 5 | | 11.00 | |  |
| 88 | 0.4 | | 0.4 | 0.1 | 0.1 | 9 | | 1 | 5.5 | 4.5 | 5 | | 12.85 | |  |
| 89 | 0.25 | | 0.25 | 0.25 | 0.25 | 3 | | 0 | 10 | 7 | 5 | | 14.50 | |  |
| 90 | 0.25 | | 0.25 | 0.25 | 0.25 | 0.5 | | 9.5 | 8.5 | 1.5 | 5 | | 16.25 | |  |
| 91 | 0.4 | | 0.1 | 0.1 | 0.4 | 0.5 | | 8 | 2 | 9.5 | 5 | | 18.00 | |  |
| 92 | 0.3 | | 0.2 | 0.3 | 0.2 | 9 | | 10 | 1 | 0 | 5 | | 19.60 | |  |
| 93 | 0.3 | | 0.2 | 0.3 | 0.2 | 10 | | 9 | 0 | 1 | 5 | | 21.40 | |  |
| 94 | 0.25 | | 0.25 | 0.25 | 0.25 | 0.5 | | 0 | 10 | 9.5 | 5 | | 22.63 | |  |
| 95 | 0.3 | | 0.3 | 0.2 | 0.2 | 10 | | 0 | 9.5 | 0.5 | 5 | | 23.10 | |  |
| 96 | 0.4 | | 0.1 | 0.1 | 0.4 | 10 | | 1 | 9 | 0 | 5 | | 23.20 | |  |
| 97 | 0.35 | | 0.35 | 0.15 | 0.15 | 0 | | 10 | 9.5 | 0.5 | 5 | | 23.58 | |  |
| 98 | 0.4 | | 0.1 | 0.1 | 0.4 | 0 | | 0.5 | 9.5 | 10 | 5 | | 24.05 | |  |
| 99 | 0.15 | | 0.35 | 0.15 | 0.35 | 4 | | 5.5 | 6.5 | 5 | 5.25 | | 0.51 | |  |
| 100 | 0.4 | | 0.1 | 0.4 | 0.1 | 4 | | 7.5 | 6.5 | 3 | 5.25 | | 2.26 | |  |
| 101 | 0.15 | | 0.15 | 0.35 | 0.35 | 6.5 | | 4 | 3 | 7.5 | 5.25 | | 4.01 | |  |
| 102 | 0.1 | | 0.1 | 0.4 | 0.4 | 0.5 | | 10 | 6.5 | 4 | 5.25 | | 5.76 | |  |
| 103 | 0.3 | | 0.2 | 0.3 | 0.2 | 3.5 | | 1.5 | 7 | 9 | 5.25 | | 7.46 | |  |
| 104 | 0.25 | | 0.25 | 0.25 | 0.25 | 4.5 | | 1 | 9.5 | 6 | 5.25 | | 9.31 | |  |
| 105 | 0.3 | | 0.2 | 0.2 | 0.3 | 5.5 | | 0 | 10.5 | 5 | 5.25 | | 11.06 | |  |
| 106 | 0.1 | | 0.4 | 0.4 | 0.1 | 8 | | 9 | 1.5 | 2.5 | 5.25 | | 12.76 | |  |
| 107 | 0.4 | | 0.1 | 0.1 | 0.4 | 9.5 | | 5 | 5.5 | 1 | 5.25 | | 14.46 | |  |
| 108 | 0.35 | | 0.15 | 0.15 | 0.35 | 10 | | 4 | 6.5 | 0.5 | 5.25 | | 16.26 | |  |
| 109 | 0.15 | | 0.35 | 0.15 | 0.35 | 8 | | 10 | 2.5 | 0.5 | 5.25 | | 18.06 | |  |
| 110 | 0.35 | | 0.15 | 0.35 | 0.15 | 10.5 | | 4 | 0 | 6.5 | 5.25 | | 19.76 | |  |
| 111 | 0.35 | | 0.15 | 0.35 | 0.15 | 10.5 | | 2.5 | 0 | 8 | 5.25 | | 21.56 | |  |
| 112 | 0.1 | | 0.4 | 0.1 | 0.4 | 3 | | 10.5 | 7.5 | 0 | 5.25 | | 23.06 | |  |
| 113 | 0.1 | | 0.1 | 0.4 | 0.4 | 7 | | 4 | 5 | 6 | 5.5 | | 0.65 | |  |
| 114 | 0.5 | | 0.25 | 0.15 | 0.1 | 6 | | 4 | 8 | 3 | 5.5 | | 2.25 | |  |
| 115 | 0.4 | | 0.1 | 0.1 | 0.4 | 4.5 | | 9.5 | 1.5 | 6.5 | 5.5 | | 4.00 | |  |
| 116 | 0.1 | | 0.4 | 0.1 | 0.4 | 10.5 | | 6.5 | 0.5 | 4.5 | 5.5 | | 5.80 | |  |
| 117 | 0.2 | | 0.4 | 0.3 | 0.1 | 1.5 | | 4.5 | 9 | 7 | 5.5 | | 7.50 | |  |
| 118 | 0.4 | | 0.1 | 0.4 | 0.1 | 7.5 | | 11 | 3.5 | 0 | 5.5 | | 9.25 | |  |
| 119 | 0.35 | | 0.15 | 0.4 | 0.1 | 2.5 | | 10.5 | 7.5 | 0.5 | 5.5 | | 11.00 | |  |
| 120 | 0.3 | | 0.3 | 0.2 | 0.2 | 6.5 | | 4.5 | 0 | 11 | 5.5 | | 12.70 | |  |
| 121 | 0.2 | | 0.3 | 0.2 | 0.3 | 0 | | 7.5 | 11 | 3.5 | 5.5 | | 14.50 | |  |
| 122 | 0.25 | | 0.25 | 0.25 | 0.25 | 4 | | 11 | 7 | 0 | 5.5 | | 16.25 | |  |
| 123 | 0.4 | | 0.1 | 0.1 | 0.4 | 10 | | 2.5 | 8.5 | 1 | 5.5 | | 18.00 | |  |
| 124 | 0.2 | | 0.3 | 0.3 | 0.2 | 7.5 | | 11 | 0 | 3.5 | 5.5 | | 19.75 | |  |
| 125 | 0.15 | | 0.15 | 0.35 | 0.35 | 4.5 | | 6.5 | 11 | 0 | 5.5 | | 21.48 | |  |
| 126 | 0.15 | | 0.15 | 0.35 | 0.35 | 11 | | 0 | 10 | 1 | 5.5 | | 23.25 | |  |
| 127 | 0.4 | | 0.4 | 0.1 | 0.1 | 6 | | 5.5 | 7.5 | 4 | 5.75 | | 0.66 | |  |
| 128 | 0.1 | | 0.4 | 0.4 | 0.1 | 8 | | 4.5 | 7 | 3.5 | 5.75 | | 2.26 | |  |
| 129 | 0.35 | | 0.15 | 0.35 | 0.15 | 3.5 | | 7 | 8 | 4.5 | 5.75 | | 4.01 | |  |
| 130 | 0.4 | | 0.1 | 0.1 | 0.4 | 7 | | 10.5 | 1 | 4.5 | 5.75 | | 5.76 | |  |
| 131 | 0.2 | | 0.2 | 0.3 | 0.3 | 2 | | 9.5 | 4 | 7.5 | 5.75 | | 7.46 | |  |
| 132 | 0.25 | | 0.25 | 0.25 | 0.25 | 1.5 | | 10 | 6.5 | 5 | 5.75 | | 9.31 | |  |
| 133 | 0.15 | | 0.15 | 0.35 | 0.35 | 11.5 | | 0 | 7 | 4.5 | 5.75 | | 11.01 | |  |
| 134 | 0.4 | | 0.4 | 0.1 | 0.1 | 2 | | 9.5 | 3 | 8.5 | 5.75 | | 12.76 | |  |
| 135 | 0.1 | | 0.1 | 0.4 | 0.4 | 5.5 | | 6 | 10 | 1.5 | 5.75 | | 14.46 | |  |
| 136 | 0.2 | | 0.3 | 0.2 | 0.3 | 11.5 | | 8 | 0 | 3.5 | 5.75 | | 16.26 | |  |
| 137 | 0.25 | | 0.25 | 0.25 | 0.25 | 4 | | 0 | 11.5 | 7.5 | 5.75 | | 18.06 | |  |
| 138 | 0.15 | | 0.35 | 0.35 | 0.15 | 0 | | 9.5 | 2 | 11.5 | 5.75 | | 19.76 | |  |
| 139 | 0.15 | | 0.35 | 0.35 | 0.15 | 3 | | 11 | 0.5 | 8.5 | 5.75 | | 21.56 | |  |
| 140 | 0.35 | | 0.15 | 0.35 | 0.15 | 0 | | 5 | 11.5 | 6.5 | 5.75 | | 23.31 | |  |
| 141 | 0.4 | | 0.1 | 0.1 | 0.4 | 6.5 | | 4 | 8 | 5.5 | 6 | | 1.00 | |  |
| 142 | 0.25 | | 0.5 | 0.15 | 0.1 | 4.5 | | 6.5 | 8.5 | 3.5 | 6 | | 2.25 | |  |
| 143 | 0.1 | | 0.4 | 0.1 | 0.4 | 10 | | 5 | 2 | 7 | 6 | | 4.00 | |  |
| 144 | 0.4 | | 0.1 | 0.4 | 0.1 | 7 | | 1 | 5 | 11 | 6 | | 5.80 | |  |
| 145 | 0.1 | | 0.3 | 0.4 | 0.2 | 7.5 | | 9.5 | 5 | 2 | 6 | | 7.50 | |  |
| 146 | 0.1 | | 0.4 | 0.1 | 0.4 | 0.5 | | 8 | 11.5 | 4 | 6 | | 9.25 | |  |
| 147 | 0.15 | | 0.1 | 0.4 | 0.35 | 11 | | 1 | 8 | 3 | 6 | | 11.00 | |  |
| 148 | 0.3 | | 0.2 | 0.3 | 0.2 | 5 | | 0.5 | 7 | 11.5 | 6 | | 12.70 | |  |
| 149 | 0.2 | | 0.2 | 0.3 | 0.3 | 0.5 | | 11.5 | 8 | 4 | 6 | | 14.50 | |  |
| 150 | 0.25 | | 0.25 | 0.25 | 0.25 | 0.5 | | 11.5 | 4.5 | 7.5 | 6 | | 16.25 | |  |
| 151 | 0.4 | | 0.1 | 0.1 | 0.4 | 10.5 | | 3 | 9 | 1.5 | 6 | | 18.00 | |  |
| 152 | 0.2 | | 0.3 | 0.2 | 0.3 | 4 | | 0.5 | 8 | 11.5 | 6 | | 19.75 | |  |
| 153 | 0.35 | | 0.15 | 0.15 | 0.35 | 11.5 | | 7 | 5 | 0.5 | 6 | | 21.48 | |  |
| 154 | 0.15 | | 0.35 | 0.35 | 0.15 | 0.5 | | 1.5 | 10.5 | 11.5 | 6 | | 23.25 | |  |
| 155 | 0.4 | | 0.1 | 0.4 | 0.1 | 6 | | 4 | 6.5 | 8.5 | 6.25 | | 1.06 | |  |
| 156 | 0.1 | | 0.1 | 0.4 | 0.4 | 8.5 | | 4 | 7.5 | 5 | 6.25 | | 2.26 | |  |
| 157 | 0.35 | | 0.15 | 0.35 | 0.15 | 4 | | 7.5 | 8.5 | 5 | 6.25 | | 4.01 | |  |
| 158 | 0.4 | | 0.4 | 0.1 | 0.1 | 7.5 | | 5 | 1.5 | 11 | 6.25 | | 5.76 | |  |
| 159 | 0.3 | | 0.3 | 0.2 | 0.2 | 4.5 | | 8 | 2.5 | 10 | 6.25 | | 7.46 | |  |
| 160 | 0.25 | | 0.25 | 0.25 | 0.25 | 7 | | 2 | 5.5 | 10.5 | 6.25 | | 9.31 | |  |
| 161 | 0.35 | | 0.35 | 0.15 | 0.15 | 5 | | 7.5 | 12 | 0.5 | 6.25 | | 11.01 | |  |
| 162 | 0.1 | | 0.4 | 0.1 | 0.4 | 9 | | 10 | 3.5 | 2.5 | 6.25 | | 12.76 | |  |
| 163 | 0.4 | | 0.1 | 0.1 | 0.4 | 2 | | 6 | 6.5 | 10.5 | 6.25 | | 14.46 | |  |
| 164 | 0.3 | | 0.2 | 0.2 | 0.3 | 4 | | 12 | 0.5 | 8.5 | 6.25 | | 16.26 | |  |
| 165 | 0.25 | | 0.25 | 0.25 | 0.25 | 4.5 | | 8 | 12 | 0.5 | 6.25 | | 18.06 | |  |
| 166 | 0.15 | | 0.35 | 0.15 | 0.35 | 12 | | 2.5 | 0.5 | 10 | 6.25 | | 19.76 | |  |
| 167 | 0.35 | | 0.15 | 0.15 | 0.35 | 1 | | 3.5 | 9 | 11.5 | 6.25 | | 21.56 | |  |
| 168 | 0.35 | | 0.15 | 0.35 | 0.15 | 12 | | 7 | 0.5 | 5.5 | 6.25 | | 23.31 | |  |
| 169 | 0.4 | | 0.1 | 0.1 | 0.4 | 7 | | 4 | 9 | 6 | 6.5 | | 1.45 | |  |
| 170 | 0.1 | | 0.25 | 0.15 | 0.5 | 4 | | 5 | 9 | 7 | 6.5 | | 2.25 | |  |
| 171 | 0.4 | | 0.1 | 0.4 | 0.1 | 7.5 | | 10.5 | 5.5 | 2.5 | 6.5 | | 4.00 | |  |
| 172 | 0.4 | | 0.1 | 0.4 | 0.1 | 5.5 | | 1.5 | 7.5 | 11.5 | 6.5 | | 5.80 | |  |
| 173 | 0.4 | | 0.1 | 0.3 | 0.2 | 5.5 | | 8 | 10 | 2.5 | 6.5 | | 7.50 | |  |
| 174 | 0.1 | | 0.4 | 0.1 | 0.4 | 12 | | 4.5 | 1 | 8.5 | 6.5 | | 9.25 | |  |
| 175 | 0.4 | | 0.35 | 0.1 | 0.15 | 8.5 | | 3.5 | 1.5 | 11.5 | 6.5 | | 11.00 | |  |
| 176 | 0.3 | | 0.2 | 0.2 | 0.3 | 7.5 | | 1 | 12 | 5.5 | 6.5 | | 12.70 | |  |
| 177 | 0.25 | | 0.15 | 0.1 | 0.5 | 12 | | 0 | 7.5 | 5.5 | 6.5 | | 14.50 | |  |
| 178 | 0.25 | | 0.25 | 0.25 | 0.25 | 12 | | 1 | 5 | 8 | 6.5 | | 16.25 | |  |
| 179 | 0.4 | | 0.1 | 0.1 | 0.4 | 11 | | 3.5 | 9.5 | 2 | 6.5 | | 18.00 | |  |
| 180 | 0.3 | | 0.2 | 0.2 | 0.3 | 1 | | 8.5 | 4.5 | 12 | 6.5 | | 19.75 | |  |
| 181 | 0.15 | | 0.15 | 0.35 | 0.35 | 5.5 | | 7.5 | 12 | 1 | 6.5 | | 21.48 | |  |
| 182 | 0.15 | | 0.15 | 0.35 | 0.35 | 1 | | 12 | 11 | 2 | 6.5 | | 23.25 | |  |
| 183 | 0.4 | | 0.1 | 0.1 | 0.4 | 6.5 | | 9.5 | 4 | 7 | 6.75 | | 1.56 | |  |
| 184 | 0.35 | | 0.15 | 0.35 | 0.15 | 6.5 | | 4 | 7 | 9.5 | 6.75 | | 2.31 | |  |
| 185 | 0.4 | | 0.1 | 0.1 | 0.4 | 5 | | 4 | 9.5 | 8.5 | 6.75 | | 3.96 | |  |
| 186 | 0.4 | | 0.4 | 0.1 | 0.1 | 5.5 | | 8 | 2 | 11.5 | 6.75 | | 5.76 | |  |
| 187 | 0.3 | | 0.2 | 0.2 | 0.3 | 8.5 | | 10.5 | 3 | 5 | 6.75 | | 7.46 | |  |
| 188 | 0.25 | | 0.25 | 0.25 | 0.25 | 2.5 | | 6 | 11 | 7.5 | 6.75 | | 9.31 | |  |
| 189 | 0.3 | | 0.3 | 0.2 | 0.2 | 7 | | 6.5 | 12 | 1.5 | 6.75 | | 11.06 | |  |
| 190 | 0.1 | | 0.1 | 0.4 | 0.4 | 9.5 | | 4 | 3 | 10.5 | 6.75 | | 12.76 | |  |
| 191 | 0.1 | | 0.4 | 0.4 | 0.1 | 6.5 | | 2.5 | 11 | 7 | 6.75 | | 14.46 | |  |
| 192 | 0.35 | | 0.35 | 0.15 | 0.15 | 2 | | 11.5 | 5.5 | 8 | 6.75 | | 16.26 | |  |
| 193 | 0.35 | | 0.15 | 0.15 | 0.35 | 11.5 | | 9.5 | 4 | 2 | 6.75 | | 18.06 | |  |
| 194 | 0.35 | | 0.15 | 0.35 | 0.15 | 12 | | 8 | 1.5 | 5.5 | 6.75 | | 19.76 | |  |
| 195 | 0.15 | | 0.15 | 0.35 | 0.35 | 4 | | 9.5 | 1.5 | 12 | 6.75 | | 21.56 | |  |
| 196 | 0.4 | | 0.1 | 0.4 | 0.1 | 1.5 | | 9 | 12 | 4.5 | 6.75 | | 23.06 | |  |
| 197 | 0.4 | | 0.4 | 0.1 | 0.1 | 6.5 | | 7.5 | 4 | 10 | 7 | | 2.00 | |  |
| 198 | 0.1 | | 0.4 | 0.1 | 0.4 | 10 | | 6 | 4 | 8 | 7 | | 2.60 | |  |
| 199 | 0.1 | | 0.1 | 0.4 | 0.4 | 3 | | 11 | 8 | 6 | 7 | | 4.00 | |  |
| 200 | 0.4 | | 0.4 | 0.1 | 0.1 | 6 | | 8 | 12 | 2 | 7 | | 5.80 | |  |
| 201 | 0.4 | | 0.1 | 0.3 | 0.2 | 6 | | 8.5 | 10.5 | 3 | 7 | | 7.50 | |  |
| 202 | 0.25 | | 0.25 | 0.25 | 0.25 | 10.5 | | 9.5 | 3.5 | 4.5 | 7 | | 9.25 | |  |
| 203 | 0.1 | | 0.4 | 0.15 | 0.35 | 2 | | 9 | 12 | 4 | 7 | | 11.00 | |  |
| 204 | 0.4 | | 0.1 | 0.4 | 0.1 | 11 | | 6.5 | 3 | 7.5 | 7 | | 12.85 | |  |
| 205 | 0.25 | | 0.25 | 0.25 | 0.25 | 5 | | 2 | 9 | 12 | 7 | | 14.50 | |  |
| 206 | 0.25 | | 0.25 | 0.25 | 0.25 | 11.5 | | 2.5 | 10.5 | 3.5 | 7 | | 16.25 | |  |
| 207 | 0.4 | | 0.1 | 0.4 | 0.1 | 2.5 | | 10 | 11.5 | 4 | 7 | | 18.00 | |  |
| 208 | 0.2 | | 0.3 | 0.3 | 0.2 | 2 | | 3 | 11 | 12 | 7 | | 19.60 | |  |
| 209 | 0.2 | | 0.2 | 0.3 | 0.3 | 11 | | 3 | 12 | 2 | 7 | | 21.40 | |  |
| 210 | 0.2 | | 0.3 | 0.3 | 0.2 | 2.5 | | 2 | 12 | 11.5 | 7 | | 23.10 | |  |
| 211 | 0.4 | | 0.1 | 0.1 | 0.4 | 2 | | 3 | 11 | 12 | 7 | | 23.20 | |  |
| 212 | 0.4 | | 0.1 | 0.4 | 0.1 | 7.5 | | 4 | 7 | 10.5 | 7.25 | | 2.16 | |  |
| 213 | 0.4 | | 0.1 | 0.4 | 0.1 | 8 | | 4 | 6.5 | 10.5 | 7.25 | | 2.56 | |  |
| 214 | 0.1 | | 0.4 | 0.4 | 0.1 | 3 | | 6.5 | 8 | 11.5 | 7.25 | | 4.06 | |  |
| 215 | 0.1 | | 0.4 | 0.4 | 0.1 | 2.5 | | 6 | 8.5 | 12 | 7.25 | | 5.76 | |  |
| 216 | 0.3 | | 0.3 | 0.2 | 0.2 | 5.5 | | 9 | 11 | 3.5 | 7.25 | | 7.46 | |  |
| 217 | 0.25 | | 0.25 | 0.25 | 0.25 | 8 | | 6.5 | 3 | 11.5 | 7.25 | | 9.31 | |  |
| 218 | 0.2 | | 0.3 | 0.3 | 0.2 | 6.5 | | 3 | 11.5 | 8 | 7.25 | | 11.06 | |  |
| 219 | 0.4 | | 0.1 | 0.1 | 0.4 | 3.5 | | 10 | 4.5 | 11 | 7.25 | | 12.76 | |  |
| 220 | 0.1 | | 0.1 | 0.4 | 0.4 | 7.5 | | 7 | 11.5 | 3 | 7.25 | | 14.46 | |  |
| 221 | 0.15 | | 0.35 | 0.15 | 0.35 | 8.5 | | 12 | 6 | 2.5 | 7.25 | | 16.26 | |  |
| 222 | 0.35 | | 0.15 | 0.15 | 0.35 | 2.5 | | 4.5 | 10 | 12 | 7.25 | | 18.06 | |  |
| 223 | 0.3 | | 0.2 | 0.2 | 0.3 | 3 | | 2.5 | 12 | 11.5 | 7.25 | | 19.86 | |  |
| 224 | 0.25 | | 0.25 | 0.25 | 0.25 | 12 | | 2.5 | 11.5 | 3 | 7.25 | | 20.31 | |  |
| 225 | 0.3 | | 0.2 | 0.3 | 0.2 | 2.5 | | 3 | 12 | 11.5 | 7.25 | | 20.76 | |  |
| 226 | 0.1 | | 0.4 | 0.4 | 0.1 | 11 | | 2.5 | 12 | 3.5 | 7.25 | | 20.86 | |  |
| 227 | 0.35 | | 0.35 | 0.15 | 0.15 | 2.5 | | 12 | 11.5 | 3 | 7.25 | | 21.21 | |  |
| 228 | 0.4 | | 0.4 | 0.1 | 0.1 | 2.5 | | 12 | 11.5 | 3 | 7.25 | | 21.66 | |  |
| 229 | 0.1 | | 0.4 | 0.4 | 0.1 | 4 | | 7 | 8 | 11 | 7.5 | | 2.65 | |  |
| 230 | 0.1 | | 0.4 | 0.1 | 0.4 | 11 | | 8.5 | 4 | 6.5 | 7.5 | | 3.25 | |  |
| 231 | 0.1 | | 0.1 | 0.4 | 0.4 | 11.5 | | 3.5 | 8.5 | 6.5 | 7.5 | | 4.00 | |  |
| 232 | 0.4 | | 0.1 | 0.4 | 0.1 | 9 | | 12 | 6 | 3 | 7.5 | | 5.85 | |  |
| 233 | 0.2 | | 0.4 | 0.3 | 0.1 | 3.5 | | 6.5 | 11 | 9 | 7.5 | | 7.50 | |  |
| 234 | 0.25 | | 0.25 | 0.25 | 0.25 | 11 | | 4 | 5 | 10 | 7.5 | | 9.25 | |  |
| 235 | 0.3 | | 0.3 | 0.2 | 0.2 | 11 | | 4 | 10.5 | 4.5 | 7.5 | | 10.95 | |  |
| 236 | 0.1 | | 0.1 | 0.4 | 0.4 | 8 | | 7 | 11.5 | 3.5 | 7.5 | | 12.85 | |  |
| 237 | 0.2 | | 0.3 | 0.3 | 0.2 | 11 | | 3.5 | 11.5 | 4 | 7.5 | | 14.50 | |  |
| 238 | 0.25 | | 0.25 | 0.25 | 0.25 | 11 | | 4 | 12 | 3 | 7.5 | | 16.25 | |  |
| 239 | 0.4 | | 0.1 | 0.4 | 0.1 | 12 | | 10.5 | 3 | 4.5 | 7.5 | | 18.00 | |  |
| 240 | 0.35 | | 0.35 | 0.15 | 0.15 | 12 | | 3 | 3.5 | 11.5 | 7.5 | | 18.98 | |  |
| 241 | 0.4 | | 0.1 | 0.4 | 0.1 | 12 | | 3.5 | 3 | 11.5 | 7.5 | | 19.40 | |  |
| 242 | 0.4 | | 0.4 | 0.1 | 0.1 | 7.5 | | 8 | 4 | 11.5 | 7.75 | | 2.86 | |  |
| 243 | 0.1 | | 0.1 | 0.4 | 0.4 | 11.5 | | 4 | 7 | 8.5 | 7.75 | | 3.26 | |  |
| 244 | 0.25 | | 0.25 | 0.25 | 0.25 | 4 | | 3.5 | 12 | 11.5 | 7.75 | | 16.06 | |  |
| 245 | 0.1 | | 0.4 | 0.4 | 0.1 | 4 | | 12 | 3.5 | 11.5 | 7.75 | | 17.26 | |  |
| 246 | 0.1 | | 0.4 | 0.4 | 0.1 | 12 | | 7 | 9 | 4 | 8 | | 4.00 | |  |
| 247 | 0.3 | | 0.1 | 0.4 | 0.2 | 7 | | 3 | 8 | 12 | 8 | | 6.00 | |  |
| 248 | 0.1 | | 0.2 | 0.4 | 0.3 | 9.5 | | 4 | 7 | 11.5 | 8 | | 7.50 | |  |
| 249 | 0.35 | | 0.15 | 0.35 | 0.15 | 5.5 | | 4 | 10.5 | 12 | 8 | | 9.18 | |  |
| 250 | 0.15 | | 0.15 | 0.35 | 0.35 | 12 | | 4 | 5 | 11 | 8 | | 11.10 | |  |
| 251 | 0.1 | | 0.1 | 0.4 | 0.4 | 8.5 | | 7.5 | 4 | 12 | 8 | | 12.85 | |  |
| 252 | 0.3 | | 0.3 | 0.2 | 0.2 | 4 | | 12 | 11.5 | 4.5 | 8 | | 14.50 | |  |

**Supplementary Table 1: Stimulus set, Experiment 1**
