# Supplementary material for: Assaying the Effect of Levodopa on the Evaluation of Risk in Healthy Humans
Source: PLoS One. 2013 Jul 3;8(7):e68177. doi: 10.1371/journal.pone.0068177 (PMC3700857; doi:10.1371/journal.pone.0068177)
Supplement: File S2 — Stimulus set, Experiment 2. Stimulus set of 252 4-outcome lotteries. Expected value of the lotteries is constant (£5.95–£6.05). Variance ranges from 1.7 to 30.9£2. Skewness ranges from −38.6 to 38.6£3. (DOCX) [file pone.0068177.s003.docx]

|  | Probabilities | | | | Amounts | | | |  |  |  |
| --- | --- | --- | --- | --- | --- | --- | --- | --- | --- | --- | --- |
| Trial | **p1** | **p2** | **p3** | **p4** | **m1** | **m2** | **m3** | **m4** | **EV** | **Var** | **Skew** |
| 1 | 0.3 | 0.2 | 0.4 | 0.1 | 6.5 | 3.5 | 7 | 6 | 6.05 | 1.72 | -2.95 |
| 2 | 0.7 | 0.1 | 0.1 | 0.1 | 6 | 9.5 | 5 | 4 | 6.05 | 1.72 | 3.13 |
| 3 | 0.6 | 0.1 | 0.2 | 0.1 | 6.5 | 7 | 6 | 1.5 | 5.95 | 2.27 | -8.60 |
| 4 | 0.55 | 0.1 | 0.1 | 0.25 | 5.5 | 10.5 | 4 | 6 | 5.975 | 2.56 | 8.44 |
| 5 | 0.5 | 0.1 | 0.1 | 0.3 | 6.5 | 6 | 1 | 7 | 6.05 | 2.92 | -12.58 |
| 6 | 0.1 | 0.1 | 0.1 | 0.7 | 11 | 4 | 6 | 5.5 | 5.95 | 3.07 | 12.07 |
| 7 | 0.15 | 0.1 | 0.55 | 0.2 | 2 | 8 | 7 | 5.5 | 6.05 | 3.40 | -8.78 |
| 8 | 0.55 | 0.1 | 0.15 | 0.2 | 5 | 4 | 10 | 6.5 | 5.95 | 3.40 | 8.78 |
| 9 | 0.35 | 0.15 | 0.3 | 0.2 | 6.5 | 4.5 | 8 | 3 | 5.95 | 3.42 | -2.95 |
| 10 | 0.2 | 0.35 | 0.15 | 0.3 | 9 | 5.5 | 7.5 | 4 | 6.05 | 3.42 | 2.95 |
| 11 | 0.4 | 0.1 | 0.4 | 0.1 | 6.5 | 6 | 7 | 0.5 | 6.05 | 3.52 | -16.72 |
| 12 | 0.2 | 0.1 | 0.1 | 0.6 | 6 | 0.5 | 8 | 6.5 | 5.95 | 3.57 | -15.23 |
| 13 | 0.1 | 0.1 | 0.2 | 0.6 | 4 | 11.5 | 6 | 5.5 | 6.05 | 3.57 | 15.23 |
| 14 | 0.1 | 0.1 | 0.5 | 0.3 | 6 | 0 | 6.5 | 7 | 5.95 | 4.02 | -20.63 |
| 15 | 0.3 | 0.1 | 0.1 | 0.5 | 6.5 | 0 | 6 | 7 | 6.05 | 4.17 | -21.69 |
| 16 | 0.1 | 0.1 | 0.6 | 0.2 | 0 | 7.5 | 6.5 | 7 | 6.05 | 4.17 | -21.61 |
| 17 | 0.1 | 0.1 | 0.1 | 0.7 | 4 | 12 | 6 | 5.5 | 6.05 | 4.17 | 20.09 |
| 18 | 0.2 | 0.1 | 0.55 | 0.15 | 6.5 | 0 | 7 | 6 | 6.05 | 4.20 | -21.65 |
| 19 | 0.1 | 0.1 | 0.7 | 0.1 | 5 | 4 | 5.5 | 12 | 5.95 | 4.27 | 21.25 |
| 20 | 0.1 | 0.45 | 0.1 | 0.35 | 4 | 5 | 12 | 6 | 5.95 | 4.45 | 21.02 |
| 21 | 0.1 | 0.1 | 0.2 | 0.6 | 3.5 | 12 | 7 | 5 | 5.95 | 5.02 | 20.39 |
| 22 | 0.25 | 0.1 | 0.55 | 0.1 | 5 | 6.5 | 7.5 | 0 | 6.025 | 5.11 | -20.36 |
| 23 | 0.15 | 0.4 | 0.1 | 0.35 | 7 | 8 | 0.5 | 5 | 6.05 | 5.12 | -14.41 |
| 24 | 0.6 | 0.1 | 0.2 | 0.1 | 7 | 5 | 2 | 9.5 | 6.05 | 5.12 | -8.78 |
| 25 | 0.6 | 0.2 | 0.1 | 0.1 | 5 | 10 | 7 | 2.5 | 5.95 | 5.12 | 8.78 |
| 26 | 0.1 | 0.15 | 0.35 | 0.4 | 11.5 | 5 | 7 | 4 | 5.95 | 5.12 | 14.41 |
| 27 | 0.2 | 0.15 | 0.4 | 0.25 | 2.5 | 5 | 8.5 | 5.5 | 6.025 | 5.16 | -2.89 |
| 28 | 0.2 | 0.15 | 0.4 | 0.25 | 9.5 | 7 | 3.5 | 6.5 | 5.975 | 5.16 | 2.89 |
| 29 | 0.15 | 0.1 | 0.6 | 0.15 | 7.5 | 6.5 | 7 | 0.5 | 6.05 | 5.50 | -24.66 |
| 30 | 0.55 | 0.2 | 0.15 | 0.1 | 5 | 5.5 | 11.5 | 4 | 5.975 | 5.54 | 24.00 |
| 31 | 0.1 | 0.15 | 0.15 | 0.6 | 4 | 11.5 | 5.5 | 5 | 5.95 | 5.57 | 24.37 |
| 32 | 0.1 | 0.1 | 0.65 | 0.15 | 7.5 | 6.5 | 7 | 0 | 5.95 | 6.30 | -30.46 |
| 33 | 0.15 | 0.6 | 0.15 | 0.1 | 7.5 | 7 | 0 | 6.5 | 5.975 | 6.36 | -30.80 |
| 34 | 0.55 | 0.1 | 0.2 | 0.15 | 5 | 4 | 5.5 | 12 | 6.05 | 6.40 | 30.07 |
| 35 | 0.15 | 0.1 | 0.6 | 0.15 | 12 | 4 | 5 | 5.5 | 6.025 | 6.44 | 30.50 |
| 36 | 0.15 | 0.1 | 0.65 | 0.1 | 12 | 5.5 | 5 | 4 | 6 | 6.48 | 30.94 |
| 37 | 0.25 | 0.5 | 0.15 | 0.1 | 7.5 | 7 | 0 | 6.5 | 6.025 | 6.49 | -31.53 |
| 38 | 0.45 | 0.15 | 0.3 | 0.1 | 7 | 0 | 7.5 | 6.5 | 6.05 | 6.55 | -31.91 |
| 39 | 0.35 | 0.35 | 0.15 | 0.15 | 7 | 7.5 | 6.5 | 0 | 6.05 | 6.57 | -31.84 |
| 40 | 0.55 | 0.15 | 0.15 | 0.15 | 5 | 5.5 | 12 | 4 | 5.975 | 6.59 | 31.13 |
| 41 | 0.1 | 0.15 | 0.15 | 0.6 | 5.5 | 4 | 12 | 5 | 5.95 | 6.62 | 31.58 |
| 42 | 0.65 | 0.15 | 0.1 | 0.1 | 5 | 12 | 3.5 | 5.5 | 5.95 | 6.70 | 31.18 |
| 43 | 0.15 | 0.2 | 0.2 | 0.45 | 12 | 4 | 5.5 | 5 | 5.95 | 6.70 | 31.33 |
| 44 | 0.55 | 0.1 | 0.1 | 0.25 | 5 | 12 | 10 | 4 | 5.95 | 6.75 | 26.46 |
| 45 | 0.5 | 0.1 | 0.25 | 0.15 | 7.5 | 5.5 | 7 | 0 | 6.05 | 6.80 | -31.49 |
| 46 | 0.2 | 0.35 | 0.15 | 0.3 | 7.5 | 8 | 7 | 2 | 5.95 | 6.80 | -14.56 |
| 47 | 0.3 | 0.2 | 0.35 | 0.15 | 10 | 4.5 | 4 | 5 | 6.05 | 6.80 | 14.56 |
| 48 | 0.1 | 0.4 | 0.1 | 0.4 | 1 | 7 | 0.5 | 7.5 | 5.95 | 6.82 | -26.36 |
| 49 | 0.2 | 0.1 | 0.4 | 0.3 | 1 | 7.5 | 8 | 6 | 5.95 | 6.82 | -20.44 |
| 50 | 0.3 | 0.2 | 0.4 | 0.1 | 6 | 11 | 4 | 4.5 | 6.05 | 6.82 | 20.44 |
| 51 | 0.2 | 0.4 | 0.25 | 0.15 | 5 | 8.5 | 2 | 7 | 5.95 | 6.85 | -8.77 |
| 52 | 0.2 | 0.15 | 0.4 | 0.25 | 7 | 5 | 3.5 | 10 | 6.05 | 6.85 | 8.77 |
| 53 | 0.25 | 0.25 | 0.25 | 0.25 | 4.5 | 8 | 9 | 2.5 | 6 | 6.88 | -2.81 |
| 54 | 0.25 | 0.25 | 0.25 | 0.25 | 4 | 3 | 9.5 | 7.5 | 6 | 6.88 | 2.81 |
| 55 | 0.1 | 0.1 | 0.1 | 0.7 | 0 | 7 | 0.5 | 7.5 | 6 | 8.30 | -35.78 |
| 56 | 0.1 | 0.2 | 0.2 | 0.5 | 0 | 6 | 3 | 8.5 | 6.05 | 8.52 | -20.47 |
| 57 | 0.3 | 0.35 | 0.2 | 0.15 | 8.5 | 2 | 7.5 | 8 | 5.95 | 8.52 | -14.56 |
| 58 | 0.15 | 0.3 | 0.35 | 0.2 | 4 | 3.5 | 10 | 4.5 | 6.05 | 8.52 | 14.56 |
| 59 | 0.1 | 0.5 | 0.2 | 0.2 | 12 | 3.5 | 6 | 9 | 5.95 | 8.52 | 20.47 |
| 60 | 0.1 | 0.15 | 0.5 | 0.25 | 3 | 0 | 8 | 7 | 6.05 | 8.55 | -32.13 |
| 61 | 0.25 | 0.5 | 0.15 | 0.1 | 5 | 4 | 12 | 9 | 5.95 | 8.55 | 32.13 |
| 62 | 0.25 | 0.15 | 0.4 | 0.2 | 9 | 7 | 6.5 | 0.5 | 6 | 8.55 | -26.33 |
| 63 | 0.4 | 0.2 | 0.25 | 0.15 | 5.5 | 11.5 | 3 | 5 | 6 | 8.55 | 26.33 |
| 64 | 0.6 | 0.1 | 0.1 | 0.2 | 6.5 | 11.5 | 8 | 1 | 6.05 | 8.57 | -8.77 |
| 65 | 0.2 | 0.15 | 0.3 | 0.35 | 8.5 | 4.5 | 9 | 2.5 | 5.95 | 8.57 | -3.00 |
| 66 | 0.2 | 0.3 | 0.35 | 0.15 | 3.5 | 3 | 9.5 | 7.5 | 6.05 | 8.57 | 3.00 |
| 67 | 0.1 | 0.6 | 0.1 | 0.2 | 4 | 5.5 | 0.5 | 11 | 5.95 | 8.57 | 8.77 |
| 68 | 0.1 | 0.1 | 0.4 | 0.4 | 12 | 11.5 | 5 | 4 | 5.95 | 8.62 | 35.93 |
| 69 | 0.2 | 0.1 | 0.4 | 0.3 | 0 | 8.5 | 7.5 | 7 | 5.95 | 9.02 | -38.63 |
| 70 | 0.2 | 0.3 | 0.4 | 0.1 | 12 | 5 | 4.5 | 3.5 | 6.05 | 9.02 | 38.63 |
| 71 | 0.2 | 0.2 | 0.15 | 0.45 | 7.5 | 0.5 | 3.5 | 8.5 | 5.95 | 10.25 | -26.38 |
| 72 | 0.2 | 0.45 | 0.2 | 0.15 | 11.5 | 3.5 | 4.5 | 8.5 | 6.05 | 10.25 | 26.38 |
| 73 | 0.15 | 0.2 | 0.4 | 0.25 | 10 | 0 | 6.5 | 7.5 | 5.975 | 10.26 | -31.94 |
| 74 | 0.15 | 0.1 | 0.6 | 0.15 | 0.5 | 3.5 | 8.5 | 3 | 5.975 | 10.26 | -20.42 |
| 75 | 0.1 | 0.5 | 0.25 | 0.15 | 4.5 | 9 | 4 | 0.5 | 6.025 | 10.26 | -14.56 |
| 76 | 0.45 | 0.25 | 0.15 | 0.15 | 2.5 | 8 | 9 | 10 | 5.975 | 10.26 | -2.87 |
| 77 | 0.45 | 0.25 | 0.15 | 0.15 | 9.5 | 4 | 2 | 3 | 6.025 | 10.26 | 2.87 |
| 78 | 0.25 | 0.1 | 0.5 | 0.15 | 8 | 7.5 | 3 | 11.5 | 5.975 | 10.26 | 14.56 |
| 79 | 0.6 | 0.15 | 0.1 | 0.15 | 3.5 | 11.5 | 8.5 | 9 | 6.025 | 10.26 | 20.42 |
| 80 | 0.2 | 0.4 | 0.25 | 0.15 | 12 | 5.5 | 4.5 | 2 | 6.025 | 10.26 | 31.94 |
| 81 | 0.5 | 0.2 | 0.15 | 0.15 | 8 | 0 | 5 | 8.5 | 6.025 | 10.29 | -37.78 |
| 82 | 0.1 | 0.35 | 0.2 | 0.35 | 1 | 8.5 | 9.5 | 3 | 6.025 | 10.29 | -8.68 |
| 83 | 0.35 | 0.1 | 0.35 | 0.2 | 3.5 | 11 | 9 | 2.5 | 5.975 | 10.29 | 8.68 |
| 84 | 0.5 | 0.2 | 0.15 | 0.15 | 4 | 12 | 3.5 | 7 | 5.975 | 10.29 | 37.78 |
| 85 | 0.15 | 0.4 | 0.1 | 0.35 | 9.5 | 9 | 3 | 2 | 6.025 | 11.94 | -8.76 |
| 86 | 0.4 | 0.35 | 0.15 | 0.1 | 3 | 10 | 2.5 | 9 | 5.975 | 11.94 | 8.76 |
| 87 | 0.4 | 0.25 | 0.1 | 0.25 | 9 | 7.5 | 4.5 | 0.5 | 6.05 | 11.95 | -32.08 |
| 88 | 0.1 | 0.15 | 0.3 | 0.45 | 8 | 6 | 1 | 9 | 6.05 | 11.95 | -26.34 |
| 89 | 0.3 | 0.15 | 0.1 | 0.45 | 11 | 6 | 4 | 3 | 5.95 | 11.95 | 26.34 |
| 90 | 0.1 | 0.4 | 0.25 | 0.25 | 7.5 | 3 | 11.5 | 4.5 | 5.95 | 11.95 | 32.08 |
| 91 | 0.25 | 0.25 | 0.1 | 0.4 | 2 | 6.5 | 0.5 | 9.5 | 5.975 | 11.99 | -14.56 |
| 92 | 0.25 | 0.2 | 0.25 | 0.3 | 4 | 10.5 | 1.5 | 8.5 | 6.025 | 11.99 | -2.77 |
| 93 | 0.25 | 0.2 | 0.25 | 0.3 | 10.5 | 1.5 | 8 | 3.5 | 5.975 | 11.99 | 2.77 |
| 94 | 0.25 | 0.4 | 0.1 | 0.25 | 10 | 2.5 | 11.5 | 5.5 | 6.025 | 11.99 | 14.56 |
| 95 | 0.1 | 0.15 | 0.2 | 0.55 | 2.5 | 7 | 0 | 8.5 | 5.975 | 12.01 | -37.84 |
| 96 | 0.15 | 0.55 | 0.2 | 0.1 | 5 | 3.5 | 12 | 9.5 | 6.025 | 12.01 | 37.84 |
| 97 | 0.15 | 0.25 | 0.15 | 0.45 | 0.5 | 10 | 1.5 | 7 | 5.95 | 12.02 | -20.37 |
| 98 | 0.25 | 0.45 | 0.15 | 0.15 | 2 | 5 | 11.5 | 10.5 | 6.05 | 12.02 | 20.37 |
| 99 | 0.15 | 0.45 | 0.25 | 0.15 | 10.5 | 2 | 9.5 | 7.5 | 5.975 | 13.64 | -2.88 |
| 100 | 0.25 | 0.45 | 0.15 | 0.15 | 2.5 | 10 | 1.5 | 4.5 | 6.025 | 13.64 | 2.88 |
| 101 | 0.4 | 0.2 | 0.1 | 0.3 | 6 | 0 | 4 | 10.5 | 5.95 | 13.67 | -14.61 |
| 102 | 0.3 | 0.2 | 0.4 | 0.1 | 1.5 | 12 | 6 | 8 | 6.05 | 13.67 | 14.61 |
| 103 | 0.25 | 0.2 | 0.35 | 0.2 | 9.5 | 0 | 8.5 | 3.5 | 6.05 | 13.70 | -32.19 |
| 104 | 0.35 | 0.15 | 0.2 | 0.3 | 9 | 0 | 9.5 | 3 | 5.95 | 13.70 | -20.42 |
| 105 | 0.2 | 0.15 | 0.3 | 0.35 | 2.5 | 12 | 9 | 3 | 6.05 | 13.70 | 20.42 |
| 106 | 0.2 | 0.2 | 0.35 | 0.25 | 12 | 8.5 | 3.5 | 2.5 | 5.95 | 13.70 | 32.19 |
| 107 | 0.15 | 0.15 | 0.25 | 0.45 | 10 | 3.5 | 0.5 | 8.5 | 5.975 | 13.71 | -26.28 |
| 108 | 0.35 | 0.2 | 0.35 | 0.1 | 10 | 8 | 2.5 | 0.5 | 6.025 | 13.71 | -8.67 |
| 109 | 0.2 | 0.1 | 0.35 | 0.35 | 4 | 11.5 | 2 | 9.5 | 5.975 | 13.71 | 8.67 |
| 110 | 0.45 | 0.15 | 0.25 | 0.15 | 3.5 | 2 | 11.5 | 8.5 | 6.025 | 13.71 | 26.28 |
| 111 | 0.25 | 0.3 | 0.3 | 0.15 | 9.5 | 0.5 | 7.5 | 8.5 | 6.05 | 13.75 | -37.90 |
| 112 | 0.3 | 0.15 | 0.3 | 0.25 | 11.5 | 3.5 | 4.5 | 2.5 | 5.95 | 13.75 | 37.90 |
| 113 | 0.2 | 0.2 | 0.45 | 0.15 | 7 | 0 | 9.5 | 2 | 5.975 | 15.31 | -32.16 |
| 114 | 0.2 | 0.2 | 0.15 | 0.45 | 5 | 12 | 10 | 2.5 | 6.025 | 15.31 | 32.16 |
| 115 | 0.2 | 0.2 | 0.5 | 0.1 | 0.5 | 2 | 9 | 10 | 6 | 15.35 | -26.18 |
| 116 | 0.1 | 0.2 | 0.2 | 0.5 | 2 | 10 | 11.5 | 3 | 6 | 15.35 | 26.18 |
| 117 | 0.1 | 0.15 | 0.2 | 0.55 | 8.5 | 1 | 0.5 | 9 | 6.05 | 15.37 | -37.92 |
| 118 | 0.55 | 0.2 | 0.15 | 0.1 | 3 | 11.5 | 11 | 3.5 | 5.95 | 15.37 | 37.92 |
| 119 | 0.2 | 0.3 | 0.25 | 0.25 | 11.5 | 6.5 | 0 | 7 | 6 | 15.38 | -20.44 |
| 120 | 0.2 | 0.25 | 0.25 | 0.3 | 0.5 | 5 | 12 | 5.5 | 6 | 15.38 | 20.44 |
| 121 | 0.3 | 0.1 | 0.25 | 0.35 | 10.5 | 6 | 7.5 | 1 | 5.975 | 15.39 | -14.41 |
| 122 | 0.3 | 0.35 | 0.1 | 0.25 | 1.5 | 11 | 6 | 4.5 | 6.025 | 15.39 | 14.41 |
| 123 | 0.25 | 0.35 | 0.2 | 0.2 | 1.5 | 8.5 | 11 | 2 | 5.95 | 15.45 | -2.80 |
| 124 | 0.35 | 0.2 | 0.2 | 0.25 | 3.5 | 10 | 1 | 10.5 | 6.05 | 15.45 | 2.80 |
| 125 | 0.4 | 0.1 | 0.2 | 0.3 | 1.5 | 5 | 9.5 | 10 | 6 | 15.45 | -8.78 |
| 126 | 0.3 | 0.2 | 0.1 | 0.4 | 2 | 2.5 | 7 | 10.5 | 6 | 15.45 | 8.78 |
| 127 | 0.15 | 0.1 | 0.4 | 0.35 | 9.5 | 10.5 | 1 | 9 | 6.025 | 17.01 | -26.28 |
| 128 | 0.15 | 0.1 | 0.35 | 0.4 | 2.5 | 1.5 | 3 | 11 | 5.975 | 17.01 | 26.28 |
| 129 | 0.15 | 0.25 | 0.2 | 0.4 | 3 | 0 | 9 | 9.5 | 6.05 | 17.05 | -38.06 |
| 130 | 0.4 | 0.2 | 0.15 | 0.25 | 2.5 | 3 | 9 | 12 | 5.95 | 17.05 | 38.06 |
| 131 | 0.5 | 0.15 | 0.2 | 0.15 | 10 | 0.5 | 4 | 1 | 6.025 | 17.09 | -14.59 |
| 132 | 0.2 | 0.5 | 0.15 | 0.15 | 8 | 2 | 11.5 | 11 | 5.975 | 17.09 | 14.59 |
| 133 | 0.1 | 0.4 | 0.2 | 0.3 | 10.5 | 10 | 1 | 2.5 | 6 | 17.10 | -3.15 |
| 134 | 0.2 | 0.3 | 0.4 | 0.1 | 11 | 9.5 | 2 | 1.5 | 6 | 17.10 | 3.15 |
| 135 | 0.1 | 0.15 | 0.4 | 0.35 | 11.5 | 8.5 | 1 | 9 | 5.975 | 17.11 | -20.29 |
| 136 | 0.15 | 0.15 | 0.35 | 0.35 | 4.5 | 8.5 | 1 | 10.5 | 5.975 | 17.11 | -8.74 |
| 137 | 0.35 | 0.15 | 0.35 | 0.15 | 11 | 7.5 | 1.5 | 3.5 | 6.025 | 17.11 | 8.74 |
| 138 | 0.35 | 0.15 | 0.4 | 0.1 | 3 | 3.5 | 11 | 0.5 | 6.025 | 17.11 | 20.29 |
| 139 | 0.4 | 0.1 | 0.35 | 0.15 | 9.5 | 10 | 0.5 | 6.5 | 5.95 | 17.12 | -32.09 |
| 140 | 0.1 | 0.15 | 0.4 | 0.35 | 2 | 5.5 | 2.5 | 11.5 | 6.05 | 17.12 | 32.09 |
| 141 | 0.25 | 0.4 | 0.2 | 0.15 | 1.5 | 10.5 | 1 | 8 | 5.975 | 18.76 | -8.72 |
| 142 | 0.25 | 0.2 | 0.4 | 0.15 | 10.5 | 11 | 1.5 | 4 | 6.025 | 18.76 | 8.72 |
| 143 | 0.3 | 0.3 | 0.25 | 0.15 | 0 | 9.5 | 10 | 4.5 | 6.025 | 18.81 | -37.85 |
| 144 | 0.1 | 0.25 | 0.2 | 0.45 | 11 | 0 | 3 | 9.5 | 5.975 | 18.81 | -26.20 |
| 145 | 0.25 | 0.1 | 0.45 | 0.2 | 12 | 1 | 2.5 | 9 | 6.025 | 18.81 | 26.20 |
| 146 | 0.3 | 0.3 | 0.25 | 0.15 | 2.5 | 12 | 2 | 7.5 | 5.975 | 18.81 | 37.85 |
| 147 | 0.2 | 0.1 | 0.3 | 0.4 | 7 | 0.5 | 1 | 10.5 | 5.95 | 18.82 | -14.66 |
| 148 | 0.1 | 0.4 | 0.3 | 0.2 | 11.5 | 1.5 | 11 | 5 | 6.05 | 18.82 | 14.66 |
| 149 | 0.3 | 0.35 | 0.15 | 0.2 | 0 | 7.5 | 6.5 | 12 | 6 | 18.83 | -20.40 |
| 150 | 0.15 | 0.3 | 0.4 | 0.15 | 1 | 1.5 | 9 | 12 | 6 | 18.83 | -2.89 |
| 151 | 0.15 | 0.4 | 0.15 | 0.3 | 11 | 3 | 0 | 10.5 | 6 | 18.83 | 2.89 |
| 152 | 0.3 | 0.15 | 0.2 | 0.35 | 12 | 5.5 | 0 | 4.5 | 6 | 18.83 | 20.40 |
| 153 | 0.25 | 0.35 | 0.2 | 0.2 | 9 | 10 | 1 | 0.5 | 6.05 | 18.90 | -31.96 |
| 154 | 0.2 | 0.25 | 0.35 | 0.2 | 11.5 | 3 | 2 | 11 | 5.95 | 18.90 | 31.96 |
| 155 | 0.5 | 0.1 | 0.2 | 0.2 | 10.5 | 1 | 0.5 | 3 | 6.05 | 20.47 | -8.68 |
| 156 | 0.2 | 0.1 | 0.2 | 0.5 | 11.5 | 11 | 9 | 1.5 | 5.95 | 20.47 | 8.68 |
| 157 | 0.35 | 0.15 | 0.4 | 0.1 | 11 | 9 | 1 | 4 | 6 | 20.50 | -3.00 |
| 158 | 0.4 | 0.35 | 0.1 | 0.15 | 11 | 1 | 8 | 3 | 6 | 20.50 | 3.00 |
| 159 | 0.45 | 0.1 | 0.2 | 0.25 | 1 | 11.5 | 10 | 9.5 | 5.975 | 20.54 | -14.55 |
| 160 | 0.25 | 0.1 | 0.45 | 0.2 | 2.5 | 0.5 | 11 | 2 | 6.025 | 20.54 | 14.55 |
| 161 | 0.2 | 0.2 | 0.35 | 0.25 | 8.5 | 7.5 | 0 | 11 | 5.95 | 20.55 | -37.47 |
| 162 | 0.35 | 0.2 | 0.2 | 0.25 | 12 | 3.5 | 4.5 | 1 | 6.05 | 20.55 | 37.47 |
| 163 | 0.2 | 0.2 | 0.45 | 0.15 | 0.5 | 6 | 10.5 | 0 | 6.025 | 20.56 | -26.21 |
| 164 | 0.2 | 0.45 | 0.25 | 0.1 | 2.5 | 9.5 | 0 | 12 | 5.975 | 20.56 | -20.14 |
| 165 | 0.45 | 0.1 | 0.2 | 0.25 | 2.5 | 0 | 9.5 | 12 | 6.025 | 20.56 | 20.14 |
| 166 | 0.45 | 0.2 | 0.2 | 0.15 | 1.5 | 11.5 | 6 | 12 | 5.975 | 20.56 | 26.21 |
| 167 | 0.4 | 0.15 | 0.35 | 0.1 | 0.5 | 11 | 9 | 10 | 6 | 20.60 | -31.95 |
| 168 | 0.35 | 0.4 | 0.1 | 0.15 | 3 | 11.5 | 2 | 1 | 6 | 20.60 | 31.95 |
| 169 | 0.4 | 0.35 | 0.1 | 0.15 | 11 | 0.5 | 2.5 | 7.5 | 5.95 | 22.15 | -8.69 |
| 170 | 0.4 | 0.1 | 0.15 | 0.35 | 1 | 9.5 | 4.5 | 11.5 | 6.05 | 22.15 | 8.69 |
| 171 | 0.2 | 0.1 | 0.4 | 0.3 | 8.5 | 1.5 | 10.5 | 0 | 6.05 | 22.17 | -37.66 |
| 172 | 0.4 | 0.3 | 0.2 | 0.1 | 1.5 | 12 | 3.5 | 10.5 | 5.95 | 22.17 | 37.66 |
| 173 | 0.3 | 0.1 | 0.2 | 0.4 | 11.5 | 7.5 | 8 | 0.5 | 6 | 22.20 | -14.70 |
| 174 | 0.15 | 0.15 | 0.45 | 0.25 | 3 | 4 | 11 | 0 | 6 | 22.20 | -3.00 |
| 175 | 0.15 | 0.45 | 0.25 | 0.15 | 8 | 1 | 12 | 9 | 6 | 22.20 | 3.00 |
| 176 | 0.3 | 0.1 | 0.2 | 0.4 | 0.5 | 4.5 | 4 | 11.5 | 6 | 22.20 | 14.70 |
| 177 | 0.1 | 0.15 | 0.4 | 0.35 | 4.5 | 11.5 | 9.5 | 0 | 5.975 | 22.26 | -32.16 |
| 178 | 0.25 | 0.15 | 0.25 | 0.35 | 6.5 | 11.5 | 10.5 | 0 | 5.975 | 22.26 | -26.16 |
| 179 | 0.1 | 0.25 | 0.15 | 0.5 | 5 | 0 | 1.5 | 10.5 | 5.975 | 22.26 | -20.54 |
| 180 | 0.5 | 0.15 | 0.25 | 0.1 | 1.5 | 10.5 | 12 | 7 | 6.025 | 22.26 | 20.54 |
| 181 | 0.15 | 0.25 | 0.25 | 0.35 | 0.5 | 5.5 | 1.5 | 12 | 6.025 | 22.26 | 26.16 |
| 182 | 0.4 | 0.35 | 0.15 | 0.1 | 2.5 | 12 | 0.5 | 7.5 | 6.025 | 22.26 | 32.16 |
| 183 | 0.1 | 0.3 | 0.3 | 0.3 | 2 | 7.5 | 0 | 12 | 6.05 | 23.87 | -8.97 |
| 184 | 0.15 | 0.4 | 0.3 | 0.15 | 8.5 | 0.5 | 12 | 6.5 | 6.05 | 23.87 | -2.97 |
| 185 | 0.15 | 0.4 | 0.3 | 0.15 | 3.5 | 11.5 | 0 | 5.5 | 5.95 | 23.87 | 2.97 |
| 186 | 0.3 | 0.3 | 0.1 | 0.3 | 0 | 4.5 | 10 | 12 | 5.95 | 23.87 | 8.97 |
| 187 | 0.15 | 0.15 | 0.35 | 0.35 | 5 | 8 | 11.5 | 0 | 5.975 | 23.94 | -14.52 |
| 188 | 0.15 | 0.35 | 0.35 | 0.15 | 4 | 12 | 0.5 | 7 | 6.025 | 23.94 | 14.52 |
| 189 | 0.5 | 0.3 | 0.1 | 0.1 | 10.5 | 0 | 0.5 | 6.5 | 5.95 | 23.97 | -32.27 |
| 190 | 0.1 | 0.3 | 0.5 | 0.1 | 5.5 | 12 | 1.5 | 11.5 | 6.05 | 23.97 | 32.27 |
| 191 | 0.25 | 0.2 | 0.15 | 0.4 | 0 | 7.5 | 0.5 | 11 | 5.975 | 23.99 | -26.48 |
| 192 | 0.4 | 0.15 | 0.2 | 0.25 | 1 | 11.5 | 4.5 | 12 | 6.025 | 23.99 | 26.48 |
| 193 | 0.1 | 0.15 | 0.35 | 0.4 | 1 | 11.5 | 0.5 | 10 | 6 | 24.03 | -20.18 |
| 194 | 0.15 | 0.35 | 0.4 | 0.1 | 0.5 | 11.5 | 2 | 11 | 6 | 24.03 | 20.18 |
| 195 | 0.1 | 0.25 | 0.25 | 0.4 | 12 | 9 | 10 | 0 | 5.95 | 24.25 | -38.41 |
| 196 | 0.4 | 0.25 | 0.25 | 0.1 | 12 | 2 | 3 | 0 | 6.05 | 24.25 | 38.41 |
| 197 | 0.1 | 0.4 | 0.2 | 0.3 | 7 | 0 | 11 | 10.5 | 6.05 | 25.57 | -37.80 |
| 198 | 0.2 | 0.1 | 0.3 | 0.4 | 1 | 5 | 1.5 | 12 | 5.95 | 25.57 | 37.80 |
| 199 | 0.45 | 0.2 | 0.15 | 0.2 | 0.5 | 11 | 11.5 | 9.5 | 6.05 | 25.60 | -20.18 |
| 200 | 0.2 | 0.2 | 0.45 | 0.15 | 1 | 2.5 | 11.5 | 0.5 | 5.95 | 25.60 | 20.18 |
| 201 | 0.4 | 0.35 | 0.15 | 0.1 | 0 | 11 | 10 | 7 | 6.05 | 25.65 | -36.80 |
| 202 | 0.5 | 0.15 | 0.25 | 0.1 | 11 | 1 | 0 | 4 | 6.05 | 25.65 | -14.90 |
| 203 | 0.1 | 0.5 | 0.15 | 0.25 | 8 | 1 | 11 | 12 | 5.95 | 25.65 | 14.90 |
| 204 | 0.1 | 0.4 | 0.15 | 0.35 | 5 | 12 | 2 | 1 | 5.95 | 25.65 | 36.80 |
| 205 | 0.1 | 0.4 | 0.4 | 0.1 | 9.5 | 11 | 0 | 6.5 | 6 | 25.65 | -32.10 |
| 206 | 0.4 | 0.1 | 0.4 | 0.1 | 1 | 5.5 | 12 | 2.5 | 6 | 25.65 | 32.10 |
| 207 | 0.1 | 0.25 | 0.45 | 0.2 | 8.5 | 12 | 0.5 | 9.5 | 5.975 | 25.69 | -8.80 |
| 208 | 0.25 | 0.45 | 0.2 | 0.1 | 0 | 11.5 | 2.5 | 3.5 | 6.025 | 25.69 | 8.80 |
| 209 | 0.35 | 0.1 | 0.45 | 0.1 | 0 | 2.5 | 11 | 8.5 | 6.05 | 25.70 | -25.93 |
| 210 | 0.15 | 0.1 | 0.25 | 0.5 | 0.5 | 0 | 1.5 | 11 | 5.95 | 25.70 | -2.98 |
| 211 | 0.25 | 0.1 | 0.5 | 0.15 | 10.5 | 12 | 1 | 11.5 | 6.05 | 25.70 | 2.98 |
| 212 | 0.1 | 0.35 | 0.45 | 0.1 | 3.5 | 12 | 1 | 9.5 | 5.95 | 25.70 | 25.93 |
| 213 | 0.3 | 0.1 | 0.25 | 0.35 | 10.5 | 1.5 | 11 | 0 | 6.05 | 26.95 | -30.17 |
| 214 | 0.35 | 0.3 | 0.25 | 0.1 | 12 | 1.5 | 1 | 10.5 | 5.95 | 26.95 | 30.17 |
| 215 | 0.35 | 0.3 | 0.1 | 0.25 | 0 | 12 | 2 | 9 | 6.05 | 27.25 | -14.54 |
| 216 | 0.1 | 0.35 | 0.25 | 0.3 | 10 | 12 | 3 | 0 | 5.95 | 27.25 | 14.54 |
| 217 | 0.4 | 0.35 | 0.1 | 0.15 | 11.5 | 0 | 9.5 | 3 | 6 | 27.28 | -8.81 |
| 218 | 0.35 | 0.4 | 0.15 | 0.1 | 12 | 0.5 | 9 | 2.5 | 6 | 27.28 | 8.81 |
| 219 | 0.25 | 0.1 | 0.25 | 0.4 | 10 | 5.5 | 12 | 0 | 6.05 | 27.42 | -20.53 |
| 220 | 0.1 | 0.4 | 0.25 | 0.25 | 6.5 | 12 | 2 | 0 | 5.95 | 27.42 | 20.53 |
| 221 | 0.1 | 0.35 | 0.45 | 0.1 | 5.5 | 0 | 11.5 | 2.5 | 5.975 | 27.46 | -2.97 |
| 222 | 0.1 | 0.1 | 0.35 | 0.45 | 9.5 | 6.5 | 12 | 0.5 | 6.025 | 27.46 | 2.97 |
| 223 | 0.1 | 0.2 | 0.25 | 0.45 | 12 | 0 | 0.5 | 10.5 | 6.05 | 27.47 | -26.31 |
| 224 | 0.25 | 0.1 | 0.2 | 0.45 | 11.5 | 0 | 12 | 1.5 | 5.95 | 27.47 | 26.31 |
| 225 | 0.4 | 0.15 | 0.15 | 0.3 | 11.5 | 0.5 | 9 | 0 | 6.025 | 28.79 | -21.32 |
| 226 | 0.3 | 0.15 | 0.15 | 0.4 | 12 | 3 | 11.5 | 0.5 | 5.975 | 28.79 | 21.32 |
| 227 | 0.45 | 0.1 | 0.35 | 0.1 | 11 | 0.5 | 0 | 10.5 | 6.05 | 28.90 | -31.21 |
| 228 | 0.45 | 0.1 | 0.35 | 0.1 | 1 | 1.5 | 12 | 11.5 | 5.95 | 28.90 | 31.21 |
| 229 | 0.25 | 0.25 | 0.1 | 0.4 | 12 | 10.5 | 3.5 | 0 | 5.975 | 29.09 | -9.00 |
| 230 | 0.25 | 0.4 | 0.1 | 0.25 | 0 | 12 | 8.5 | 1.5 | 6.025 | 29.09 | 9.00 |
| 231 | 0.15 | 0.4 | 0.3 | 0.15 | 5 | 0 | 12 | 11 | 6 | 29.10 | -3.00 |
| 232 | 0.15 | 0.15 | 0.3 | 0.4 | 7 | 1 | 0 | 12 | 6 | 29.10 | 3.00 |
| 233 | 0.45 | 0.15 | 0.1 | 0.3 | 11.5 | 0.5 | 7.5 | 0 | 6 | 29.18 | -14.55 |
| 234 | 0.15 | 0.1 | 0.3 | 0.45 | 11.5 | 4.5 | 12 | 0.5 | 6 | 29.18 | 14.55 |
| 235 | 0.15 | 0.1 | 0.3 | 0.45 | 12 | 10 | 10.5 | 0 | 5.95 | 29.27 | -26.67 |
| 236 | 0.15 | 0.1 | 0.45 | 0.3 | 0 | 2 | 12 | 1.5 | 6.05 | 29.27 | 26.67 |
| 237 | 0.1 | 0.35 | 0.45 | 0.1 | 10.5 | 11 | 0 | 11.5 | 6.05 | 30.00 | -32.20 |
| 238 | 0.1 | 0.45 | 0.1 | 0.35 | 0.5 | 12 | 1.5 | 1 | 5.95 | 30.00 | 32.20 |
| 239 | 0.15 | 0.25 | 0.45 | 0.15 | 11.5 | 11 | 0 | 10.5 | 6.05 | 30.02 | -31.83 |
| 240 | 0.45 | 0.25 | 0.15 | 0.15 | 12 | 1 | 0.5 | 1.5 | 5.95 | 30.02 | 31.83 |
| 241 | 0.2 | 0.15 | 0.2 | 0.45 | 11.5 | 11 | 10.5 | 0 | 6.05 | 30.05 | -31.46 |
| 242 | 0.2 | 0.15 | 0.45 | 0.2 | 1.5 | 1 | 12 | 0.5 | 5.95 | 30.05 | 31.46 |
| 243 | 0.2 | 0.1 | 0.45 | 0.25 | 12 | 11.5 | 0 | 10 | 6.05 | 30.42 | -25.93 |
| 244 | 0.25 | 0.1 | 0.45 | 0.2 | 2 | 0.5 | 12 | 0 | 5.95 | 30.42 | 25.93 |
| 245 | 0.35 | 0.1 | 0.1 | 0.45 | 12 | 8 | 10 | 0 | 6 | 30.80 | -14.40 |
| 246 | 0.35 | 0.45 | 0.1 | 0.1 | 0 | 12 | 4 | 2 | 6 | 30.80 | 14.40 |
| 247 | 0.1 | 0.45 | 0.15 | 0.3 | 11 | 0 | 9 | 12 | 6.05 | 30.85 | -20.48 |
| 248 | 0.3 | 0.15 | 0.1 | 0.45 | 12 | 11 | 7 | 0 | 5.95 | 30.85 | -8.92 |
| 249 | 0.45 | 0.15 | 0.1 | 0.3 | 12 | 1 | 5 | 0 | 6.05 | 30.85 | 8.92 |
| 250 | 0.3 | 0.15 | 0.45 | 0.1 | 0 | 3 | 12 | 1 | 5.95 | 30.85 | 20.48 |
| 251 | 0.4 | 0.4 | 0.1 | 0.1 | 12 | 0 | 3 | 9.5 | 6.05 | 30.92 | -3.05 |
| 252 | 0.1 | 0.1 | 0.4 | 0.4 | 2.5 | 9 | 0 | 12 | 5.95 | 30.92 | 3.05 |

**Supplementary Table 2: Stimulus set, Experiment 2**
